# Under-Five Mortality in High Focus States in India: A District Level Geospatial Analysis

**DOI:** 10.1371/journal.pone.0037515

**Published:** 2012-05-18

**Authors:** Chandan Kumar, Prashant Kumar Singh, Rajesh Kumar Rai

**Affiliations:** 1 Department of Humanities and Social Sciences, Indian Institute of Technology (IIT), Roorkee, Uttarakhand, India; 2 International Institute for Population Sciences, Mumbai, Maharashtra, India; 3 Tata Institute of Social Sciences, Mumbai, Maharashtra, India; Tehran University of Medical Sciences, Iran (Islamic Republic of)

## Abstract

**Background:**

This paper examines if, when controlling for biophysical and geographical variables (including rainfall, productivity of agricultural lands, topography/temperature, and market access through road networks), socioeconomic and health care indicators help to explain variations in the under-five mortality rate across districts from nine high focus states in India. The literature on this subject is inconclusive because the survey data, upon which most studies of child mortality rely, rarely include variables that measure these factors. This paper introduces these variables into an analysis of 284 districts from nine high focus states in India.

**Methodology/Principal Findings:**

Information on the mortality indicator was accessed from the recently conducted Annual Health Survey of 2011 and other socioeconomic and geographic variables from Census 2011, District Level Household and Facility Survey (2007–08), Department of Economics and Statistics Divisions of the concerned states. Displaying high spatial dependence (spatial autocorrelation) in the mortality indicator (outcome variable) and its possible predictors used in the analysis, the paper uses the Spatial-Error Model in an effort to negate or reduce the spatial dependence in model parameters. The results evince that the coverage gap index (a mixed indicator of district wise coverage of reproductive and child health services), female literacy, urbanization, economic status, the number of newborn care provided in Primary Health Centers in the district transpired as significant correlates of under-five mortality in the nine high focus states in India. The study identifies three clusters with high under-five mortality rate including 30 districts, and advocates urgent attention.

**Conclusion:**

Even after controlling the possible biophysical and geographical variables, the study reveals that the health program initiatives have a major role to play in reducing under-five mortality rate in the high focus states in India.

## Introduction

India has the world's highest percentage (21%) of under-five deaths, estimated at 1726000 in 2009. The country managed to reduce the under-five mortality rate (U5MR) from 118 per 1000 live births in 1990 to 66 per 1000 live births in 2009. The average annual rate of decline at 3.1% was considered insufficient to achieve Millennium Development Goal (MDG) 4 that targets minimizing under-five mortality to 39 per 1000 live births by 2015 [1]. The north-south variation in child mortality in India is reflected in literature [2], [3] where some of the north Indian states such as Rajasthan, Uttar Pradesh, Bihar, Orissa, Chhattisgarh and Madhya Pradesh persistently performed poorly in health care [4]. On account of the unacceptably high fertility and mortality indicators, the eight Empowered Action Group (EAG) states (Bihar, Chhattisgarh, Jharkhand, Madhya Pradesh, Orissa, Rajasthan, Uttarakhand, Uttar Pradesh and Assam), which account for about 48% of India's population, are designated as “High Focus States” by the Government of India. The U5MR in Uttar Pradesh (94 per 1000 live births), Madhya Pradesh (89 per 1000 live births), Orissa (82 per 1000 live births), Assam and Bihar (77 and 78 per 1000 live births) are almost similar to the U5MR in some African countries – Djibouti (94 per 1000 live births), Zimbabwe (90 per 1000 live births), Kenya (84 per 1000 live births), Sao Tome and Principe (78 per 1000 live births) respectively [1].

Based on the district level U5MR that has been made available recently by the Annual Health Survey (AHS) 2011 [5], we assess the levels of under-five mortality and its spatial pattern in these high focus states in India. Using exploratory spatial data analysis (ESDA) and spatial econometric methods, this paper examines if, when controlling for biophysical and geographical variables, socioeconomic and health programs related indicators help to explain variation in U5MR across 284 districts in 9 high focus group states. We also intend to identify some of the critical districts with high under-five mortality in order to prioritize the implementation of program initiatives.

A number of studies attempted to demonstrate the indirect causes of childhood illnesses, but none of them proved as influential in formulating public policy as the framework proposed by Moseley and Chen [6]. According to them, socioeconomic factors such as education and income affect disease incidence and outcomes through five broad groups of “proximal determinants” of child survival: maternal factors, nutrient deficiency, environmental contamination, injury and personal illness control characterized by the availability of health services and the capacity to use them [7]. The importance of these factors has been repeatedly confirmed in the reports issued by the World Health Organization (WHO), United Nations Children's Fund (UNICEF) and United Nations Population Fund (UNFPA). Coupled with early motherhood, poor nutrition including anemia, low use of antenatal care and skilled delivery care potentially aggravates the chances of child deaths [8]–[10]. India with nearly 60 million malnourished children and more than 50% suffering from anemia was estimated to be amongst the highest in the world for under-five child deaths [11], [12]. A growing body of evidence suggests that diarrhea, malaria – the diseases that are responsible for child mortality in developing countries, are results of climate change [13], [14]. Changes in precipitation and the warming pattern are likely to affect the quality and quantity of water supplies, thus compounding the impact of poor water and sanitation, as well as malnutrition to the poorest in particular [15], [16], [17].

Despite several policies and program provisioning, the accessibility, availability and affordability of child health care services remain a challenge to the Indian health care system [18]. Low child immunization accelerates the probability of childhood deaths in India [4]. In order to reduce child mortality, the Government of India launched an ambitious National Rural Health Mission (NRHM) in April 2005, where the Child Health Program (CHP) comprehensively integrated interventions that improve child health and address factors contributing to infant and under–five mortality [19]. The major components of CHP are – the establishment of Newborn Care facilities and Facility Based Integrated Management of Neonatal and Childhood Illnesses (F-IMNCI); *Navjaat Shishu Suraksha Karyakram*; Integrated Management of Neonatal and Childhood Illnesses (IMNCI) and Pre-Service IMNCI; home based care of newborns, universal immunization, early detection and appropriate management of Acute Respiratory Infections (ARI), diarrhea and other infections coupled with other supplementation and school health programs. However, the main barrier to extensive coverage of integrated packages for health of mothers, neonates and children in most countries including India [18] is inadequate operational management, especially at the district level [20]. The socioeconomic inequalities in child health care and health status are highlighted in demographic and public health literature [4], [18], [21], [22].

Public health research has focused on understanding the health of the population in different geographical regions or space (using geospatial analysis) as possible contextual factors [23], [24], [25], [26], [27], [28], [29], [30], [31], [32]. Increasingly sophisticated geographic information systems have made this advancement possible. Using geospatial analysis, it is now possible to account for the effect of spatial diffusion in particular health or socio-economic parameters and a number of contiguous and contagious biophysical and geographical variables. It becomes an essential tool, when there is presence of spatial autocorrelation [33], [34], [35] in the variables, which violates Gauss-Markov assumptions (leading to unreliable statistical inference while using ordinary least squares {OLS} regression technique) [36]. Examples of biophysical and geographic factors often cited in literature include rainfall, temperature [15]–[17], [37], productivity of agricultural lands [38], distance to urban areas [37], malaria endemicity [28], [39], frequency of drought, topography and market access through road networks [39], [40]. Several studies have highlighted the importance of such variables in explaining the variations in infant and child mortality [37], [41], [42], [43], [44], [45].

However, the literature on this subject, especially in the Indian context, is inconclusive because the survey data, upon which most studies of child mortality rely, rarely include variables that measure these factors. Here, this study adopts a comprehensive approach of geospatial analysis to explain spatial variation/clustering in U5MR, and accounts for biophysical and geographical correlates in the high focus states of India. Although, poor performance in socioeconomic and health indicators among high focus states has been documented [46], [47], [48], [49], to our knowledge, hardly any study in India ever has made a conclusive effort to demonstrate spatial pattern/clustering in under-five mortality at the district level, and considered biophysical and geographic variables such as temperature, rainfall, productivity of agricultural lands, and market access through road networks in child mortality analysis. The findings of this study are expected to provide an improved understanding of district level under-five mortality and its correlates, which may in turn help focus on reducing child deaths in certain districts in high focus group states.

## Methods

### Ethics Statement

The study is based on the data available in the public domain to use; therefore, no ethics statement is required for this work.

### Data

The study focuses on nine high focus states in India - Bihar, Chhattisgarh, Jharkhand, Madhya Pradesh, Orissa, Rajasthan, Uttarakhand, Uttar Pradesh and Assam, consisting of 284 districts. The district level data for all nine states were culled from the recently concluded Annual Health Survey (AHS) - 2011, Census - 2011, District Level Household and Facility Survey (DLHS) - 3 (2007–08), India Meteorological Department, Ministry of Agriculture, Department of Economics and Statistics Division, and Statistical Abstract of concerned states. A detailed description of data sources with selected variables are presented in **Table 1**.

**Table 1:** Description of Variables.

Variable description	Sources
**Dependent Variable**	
Under-5 Mortality Rate (Per 1000 live births)	AHS (2011)
**Biophysical Variables**	
Average Annual Temperature (°C)	SA, Various States (Web Services)
Annual Rainfall (in mm)	HD, IMD (2006–10)
**Agricultural Variable**	
Yield of total food grains (quintal./hectare)	DES, MoA, GoI
**Geographic Variables**	
Proportion of District within 2 km of a road	Calculated using GIS
Population Density (persons/sq. km)	Census, 2011
**Health Indicators**	
Coverage Gap Index (A Composite Index based on reproductive and child health care)	DLHS- 3 (2007–08)
Proportion (%) of CHCs with Low Birth Weight Management	DLHS- 3 (2007–08)
No. of Newborn Care provided in PHCs	DLHS- 3 (2007–08)
Proportion (%) of HHs having Knowledge about Malaria Prevention	DLHS- 3 (2007–08)
**Socioeconomic Variables**	
Per Capita Gross District Domestic Product ( 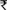 ) [GDDPPC]	DESD, Various State Governments
Proportion (%) of HHs having BPL Cards	DLHS- 3 (2007–08)
Proportion (%) of SC/ST Population	DLHS- 3 (2007–08)
Proportion (%) of HHs having access to Piped Water	DLHS- 3 (2007–08)
Female Literacy (%)	Census, 2011
Proportion (%) of Urban Population	Census, 2011

AHS = Annual Health Survey; SA = Statistical Abstract; HD, IMD = Hydromet Division, India Meteorological Department; DES, MoA, GoI = The Directorate of Economics & Statistics (DES), Ministry of Agriculture, Govt. of India; GIS = Geographical Information System; DLHS = District Level Household and Facility Survey; DESD = The Directorate of Economics & Statistics Division; CHC = Community Health Center; PHC = Primary Health Center; SC/ST = Scheduled Castes/Scheduled Tribes.

Realizing the need for decentralized district-based health planning in India, the Office of the Registrar General, Government of India implemented the Annual Health Survey (AHS) in all the 284 districts (as per 2001 Census) in the eight Empowered Action Group States and Assam (for a three-year period) during the Eleventh Five Year Plan period (2007–2012). These nine states, which account for about 48% of the total population in the country, are the high focus states in view of their relatively high fertility and mortality indicators. The fieldwork for Baseline Survey was carried out from July 2010 to March 2011. For the first time in the country, the survey provides district level estimates on a set of child mortality indicators like infant mortality rate, under-five mortality rate (U5MR), neonatal mortality rate, and post-neonatal mortality rate in the mentioned high focus states. Further details of data collection and management procedures are available on the survey website [5]. The present study utilized these district level estimates of U5MR provided by AHS 2011 conducted during 2010–11 in nine high focus states in India.

The 15th Indian National Census was conducted by the Office of the Registrar General, Government of India between February 9 and 28, 2011 (population enumeration phase). Spread across 35 states and union territories of India, the Census covered 640 districts and 5767 *talukas* [50]. About 2.7 million officials visited households in 7935 towns and 640867 villages, classifying the population according to gender, religion, education and occupation [51]. The population enumeration schedule collected information on a wide range of demographic and socioeconomic indicators of the household and the individual. The district level information on female literacy, population density and level of urbanization from Census 2011 [50] were used as covariates in the analysis.

Other socioeconomic and health indicators were collected from the District Level Household and Facility Survey (DLHS-3) conducted by the International Institute for Population Sciences (IIPS), Mumbai, India [52]. DLHS-3 is one of the largest ever demographic and health surveys carried out in India, with a sample size of over seven hundred thousand households covering 601 districts of the country. It provides estimates on maternal and child health, family planning and other reproductive health indicators. For the first time, a population-linked facility survey was conducted in DLHS-3. The health facility questionnaires contained information on human resources, infrastructure and services. At the district level, all Community Health Centres (CHC) and District Hospitals were covered. Further, all Sub-Centres and Primary Health Centres (PHC), which were expected to serve the population of the selected Primary Sampling Unit (PSU), were also covered in the survey.

The biophysical data - rainfall and temperature was accessed from Hydromet Division, India Meteorological Department, and Statistical Abstract of selected states. India Meteorological Department (IMD) established in 1875 is the National Meteorological Service in India and the principal government agency in all matters relating to Meteorology, Seismology and allied subjects. For administrative control and technical operations, six Regional Meteorological Centres (RMCs) function with their headquarters at Kolkata, Chennai, Guwahati, Mumbai, Nagpur and New Delhi. In addition, the district wise agricultural indicator was collected from the Ministry of Agriculture, Government of India.

### Measures

The outcome variable of this study is the under-five mortality rate (U5MR), which is regarded as the most sought after indicator to assess the progress of the Millennium Development Goal (MDG)-4. The U5MR was collected for 284 districts in nine states from AHS - 2011. The under-five mortality is the probability (^5^q^0^) that a child born in a specific year or period will die before reaching the age of five, subject to current age specific mortality rates. It is expressed as a rate per 1000 live births. The study assesses the variation in U5MR (2010–11) through a set of independent predictors. These predictors include biophysical, agriculture, and geographic variables apart from health and socioeconomic indicators of the sample districts.

**Table 1** presents a brief description of all the variables selected for this study from different sources. The biophysical variables include temperature and rainfall; however, the average elevation of the districts being highly correlated with temperature, was not considered in the study. The average annual temperature of the district represented in degree Celsius (°C) varies in its reference period from 2007 to 2009. The annual rainfall (in mm) was averaged from the IMD estimates for five years (2006–10). The yield of total food grains (quintal/hectare) in the district surrogates the agricultural productivity, and relates to the period 2007–08. However, in the absence of recent agricultural data for 16 districts in Chhattisgarh, the estimates of food grain productivity relate to the period, 2002–03.

Geographical variables include road accessibility and population density in the district. Road accessibility is measured as the proportion of the district's territory that is within 2 km of a paved or improved road. The 2 km buffered zone around the metalled road network within the district boundary was calculated using Geographic Information System (GIS). The average population density of the district represents the number of persons per square km. of the district area, and the estimate relates to 2011.

The health indicators comprise an overall coverage of reproductive and child health services, selected health infrastructure, and household level knowledge about the prevention of malaria in the district. A range of reproductive and child health interventions is used to develop a composite measure of health system coverage to compare health system performance across districts. A detailed description of the variables selected for constructing the Coverage Gap Index (CGI) along with their definitions is given in **Appendix S1**. The CGI is a measure of overall coverage and health system strength [53]. This is a composite index assimilating a set of four intervention areas, which are presented along the continuum of care [20], a major theme of the 2008 Countdown: family planning, maternal and newborn care, immunization, and treatment of sick children. In each intervention area, one to three indicators have been selected. These coverage indicators are consistent with those used in the 54 Countdown countries in 2008, except that BCG has been added to the immunization area. In the family planning domain, we selected the contraceptive prevalence rate for the modern methods of contraception - the percentage of women aged 15–49 years currently married or in union who are using (or whose partner is using) a modern contraceptive method. In surveys without data for the need for family planning, the indicator can be estimated from the widely available data for contraceptive prevalence rate. For high contraceptive prevalence rates (exceeding 68%), the estimated need satisfied was kept at 100% [53].

All the measures to compute CGI were estimated at the district level using DLHS-3 (2007–08) data. The Cronbach's α reliability coefficients were calculated to ascertain the internal consistency of the items (the four intervention areas) in relation to the underlying construct. Cronbach's α reliability coefficient has a theoretical value of between 0 and 1, and values exceeding 0·7 for the coefficient are regarded as acceptable [54]. Item analysis aims to improve the reliability of the index by identifying items that are poorly correlated with other items [55]. Cronbach's α reliability coefficient was 0·853 for the full set of eight coverage indicators. No item was removed. Equal weight was assigned to all four intervention areas and within each intervention area. The only exception was DPT3 coverage, which was given a weight of 2, since it involved multiple contacts with the health services and correlated highly with other vaccinations such as those for poliomyelitis and Haemophilus influenza B [53]. The formula to calculate the coverage gap index is
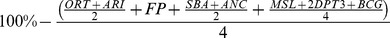
where ORT = oral rehydration therapy; ARI = acute respiratory infection; FP = family planning; SBA = skilled birth attendance; ANC = antenatal care; MSL = measles vaccination; and DPT3 = three doses of diphtheria, pertussis, and tetanus vaccine.

The result is presented as a measure of the gap between maximum and actual coverage for several reasons. First, monitoring progress towards reduction of the coverage gap becomes a more meaningful comparison once coverage of interventions is over 50%. Second, a gap measure allows for the introduction of new interventions, such as malaria or micronutrient interventions, in a more meaningful way than coverage allows; increasing the number of interventions that health systems need to deliver will expand the gap between ideal and actual coverage for all the interventions combined. Third, theoretically, the goal might not be 100% coverage for some interventions, and a gap measure allows the user to define lower goals as a target. Fourth, it clearly distinguishes the aggregate index from ordinary intervention coverage measures [53].

Other health indicators included were the percentage of Community Health Centers (CHCs) with low birth weight management system, number of newborn care provided in Primary Health Centers (PHCs), and the percentage of households with knowledge about prevention of malaria in each selected district. The first two indicators were estimated using DLHS-3 Facility Survey, and the latter using DLHS-3 household data.

Socioeconomic indicators include per capita gross district domestic product, percentage of households having BPL (Below Poverty Line) cards, percentage of Scheduled Castes (SCs)/Scheduled Tribes (STs) population, percentage of households having access to piped water, female literacy (%), and level of urbanization (%) in each selected district. Most variables approximated a normal distribution, but five variables required log transformations owing to skewed distributions. These included population density, per capita gross district domestic product, percentage of SC/ST population, and percentage of households having access to piped water. The poor are identified by a Below Poverty Line (BPL) Survey carried out by the District Rural Development Authority (DRDA) of each state with guidelines from the Ministry of Rural Development, Government of India [56].

### Analytical Approach

The outcome variable (U5MR) and the predictors were diagnosed using descriptive statistics of 284 sample districts. Local small-area variation in mortality lends itself readily to investigation via spatial analysis, the functions of which include detecting spatial patterns in data and formulating hypotheses based on the geography of the data [57]. We used the ArcGIS software package to generate maps for all the indicators, and assessed the spatial dependence in district level estimates using Moran's *I* Index (Global) value [58]. In general, a Moran's Index value near +1 indicates clustering, while an index value near −1 indicates dispersion.

The spatial pattern of U5MR across sample districts was analyzed using two different spatial weights. Spatially contiguous weights are generally computed in two ways: (a) rook's weight (uses common boundaries to define neighbour), and (b) queen's weight (includes all common points - boundaries and vertices) [29], [59]. We used both the weights to manifest the spatial clustering and outliers in the outcome variable (U5MR) using Anselin Local Moran's *I* statistics (in ArcGIS) and LISA (Local Indicators of Spatial Autocorrelation, in GeoDa), where ArcGIS used polygon contiguity (first order) or queen's weight, and the rook's weight was used in GeoDa [60], [61]. We calculated the significance of the local Moran's *I* using a randomisation test on the Z-score with 9999 permutations to achieve highly significant values [35]. We also used Getis-Ord Gi* statistics [62], [63] using ArcGIS to assess the hotspots in the outcome variable and to compare it with the local Moran's *I* statistics. The details of the different methods of exploratory spatial data analysis (ESDA) used in this paper and discussion on spatial weights are presented in **Appendix S2**.

Having known the presence of spatial dependence in the outcome and predictor variables, we realized that the assumption of independent observations and errors of classical statistical models might be violated. Therefore, we applied and compared three regression models to examine the relationship between the outcome variable and a set of predictors: ordinary least square (OLS), spatial lag model (SLM) [64], [65], and spatial error model (SEM) [66], [67]. Given a particular choice of the spatial weights matrix, the latter two models are important and distinct in ways spatial interaction is modeled in spatial regression analysis. Spatial regression methods capture spatial dependency in regression analysis, avoiding statistical problems such as unstable parameters and unreliable significance tests, and provide information on spatial relationships among the variables involved. While the OLS regression model takes the form:
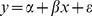
the spatial lag model (also called Spatial Auto-Regressive model) takes the form:
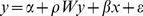
and the spatial error model takes the form:
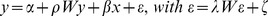
where, *y* represents the U5MR, *α* is an intercept, *β* is the vector of regression parameters, *x* is the matrix of exogenous explanatory variables, ε is the vector of regression disturbances (i.i.d), *Wy* the spatial lag term, *ρ* is the spatial autoregressive parameter of *Wy* (which is estimated for the model as a whole), and λ is the coefficient of spatially lagged autoregressive errors, *Wε*. Errors in 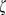 are independently distributed, and *W* is spatial weight.

A correlation matrix was computed to assess the association between the outcome variable and predictors before applying the multivariate OLS and spatial models. We used GeoDa 0.9.5-i (Beta) software to compute spatial regression models using rook's weight. Consequently, we also employed Bivariate LISA [23], [24], [33] to assess the spatial interdependence between the outcome variable and a significant and important predictor variable.

## Results

### Sample Characteristics

**Table 2** presents descriptive statistics for each of the selected variables, including the global Moran's *I* Index value. The outcome variable, U5MR ranges from 24 to 145 per 1000 live births in 284 sample districts in 9 states of India. The average annual temperature across the sample districts demonstrated less variation (SD = 2.2°C) with a mean temperature of about 25°C. The average annual rainfall during 2006–10 ranges from a minimum of 209 mm to a maximum of 3285 mm with a mean of 990 mm and a SD of 524 mm. Foodgrains productivity in the region ranges from a minimum of about 2 quintal/hectare to a maximum of 35 quintal/hectare. The proportion of district area within 2 km from the metalled road was estimated at 0.2% (lowest) to 53% (highest). The least population density of 17 persons/sq. km was recorded in Jaisalmer, Rajasthan, while the most densely populated district in the region was Ghaziabad, UP with 2383 persons/sq. km in 2011. The coverage gap index ranges from the least gap in Indore (33%), Madhya Pradesh to the maximum gap in Bahraich (79%), Uttar Pradesh with a mean of 56% (SD = 10%) in the region. There was no system of low birth weight management (LBWM) in place in the Community Health Centres (CHCs) of 77 districts of the region. The average proportion of CHCs that had LBWM system care was 34% (SD = 30%). Similarly, in 7 districts of Orissa, a state with high U5MR, and 2 districts of Uttarakhand, not a single case of newborn care was provided in Primary Health Centres (PHCs) in the region. The average proportion of households with knowledge about malaria prevention was about 81% (SD = 18%) in the region.

**Table 2:** Descriptive Statistics and Moran's *I* value of Variables.

Variables	Min.	Max.	Mean	SD	Moran's I^a^
U5MR (per 1000 live births)	24	145	81	20	0.439
Average Annual Temperature (°C)	9.6	27.3	25.1	2.2	0.849
Annual Rainfall (mm)	209	3285	990	524	0.743
Foodgrain Yield (quintal./hectare)	1.7	34.5	15.9	6.6	0.617
% Area of District within 2 km from Road	0.2	52.7	34.3	7.6	0.484
Population Density (person/sq. km)	17	2383	568	437	0.690
Coverage Gap Index [CGI]	32.5	79.2	56.2	10.4	0.634
% CHC with Low Birth Weight Management	0.0	100.0	33.9	29.5	0.169
Number of newborn care provided in PHC	0	4437	384	605	0.337
Knowledge about Malaria Prevention	8.8	99.62	80.5	17.5	0.455
Per Capita Gross District Domestic Product ( 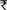 ) [GDDPPC]	3636	56029	13620	6674	0.464
% HH having BPL Card	4.3	69.6	32.9	14.8	0.640
% SC/ST Population	9.6	86.5	32.8	16.8	0.603
% HH with Piped Water	0.0	73.0	8.9	10.7	0.551
Female literacy (%)	32.2	82.1	58.0	9.6	0.492
% Urban Population	3.4	80.8	18.7	13.7	0.194

N = 284 (Districts).

a All Moran's I value is significant at p<0.01.

An average per capita gross district domestic product of 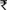 (Indian National Rupee) 13620 (SD = 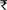 6674) was recorded in the region, ranging from 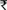 3636 (in Sheohar, Bihar) to 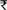 56023 (in Korba, Chhattisgarh). Another surrogate indicator of economic status of average people in the district is the percentage of households possessing a BPL card, which was the least (about 4%) in Agra (Uttar Pradesh), and the maximum (about 70%) in Bastar district of Chhattisgarh. The highest proportion of SC/ST population was reported in Malkangiri, Orissa with an average of 33% (SD = 17%) in the region. Almost 50 out of 284 districts in the region reported below 1% households having access to piped water in dwelling or yard/plot, public tap and bottled water facility. In 2011, the highest level of female literacy and urbanization in the region was reported in Khordha (82%), Orissa and Bhopal (81%), Madhya Pradesh respectively. However, Dantewada (32%), Chhattisgarh and Shrawasti (3%), Uttar Pradesh reported the lowest level of female literacy and urbanization. The descriptive statistics of sample districts by each state separately can be seen in **Appendix S3**.

The correlation matrix (**Table 3**) manifests a number of significant correlations between the independent variables and the U5MR. Temperature (+), rainfall (−), coverage gap index (+), knowledge about malaria prevention (−), per capita gross district domestic product (−), households having BPL cards (+), households having access to piped water (−), urbanization (−), and female literacy (−) show significant correlation with the outcome variable. The coverage gap index has the maximum value of *r* (0.443; p = 0.000), followed by temperature, per capita gross district domestic product and level of urbanization. Surprisingly, accessibility to roads is positively related with U5MR, which reflects that the mortality rate is high in more accessible districts with a high population density. Other highly correlated set of variables are population density, accessibility to road, yield of foodgrains, and proportion of SC/ST population, where the latter is negatively correlated with the other three indicators. Variables like agricultural productivity (represented by foodgrain yield), accessibility to road, GDDP per capita, proportion of SC/ST population, and households having access to piped water were dropped in the final multivariate analysis, as they were correlated with some other variables in the analysis and not considered best fit.

**Table 3:** Correlation Matrix of all Variables.

	Variable	1	2	3	4	5	6	7	8	9	10	11	12	13	14	15	16
1	U5MR	1.000															
2	Average Annual Temperature (°C)	0.335#	1.000														
3	Annual Rainfall (mm)	−0.204#	−0.223#	1.000													
4	Foodgrain Yield (quintal./hectare)$	0.049	0.086	−0.271#	1.000												
5	District within 2 km from Road$	0.194#	0.353#	−0.189#	0.246#	1.000											
6	Population Density (person/sq. km) (log)	0.112	0.292#	−0.124*	0.508#	0.512#	1.000										
7	Coverage Gap Index (CGI)	0.443#	0.121*	−0.197#	0.243#	0.323#	0.432#	1.000									
8	CHC with Low Birth Weight Management	−0.033	−0.025	0.068	−0.080	−0.190#	−0.257#	−0.235#	1.000								
9	Number of newborn care provided in PHC (log)	0.009	0.324#	−0.010	−0.005	0.112	0.204#	0.061	0.066	1.000							
10	Knowledge about Malaria Prevention$	−0.215#	0.089	0.087	−0.090	−0.287#	−0.364#	−0.559#	0.144*	−0.037	1.000						
11	Per Capita GDDPPC (log)$	−0.327#	−0.204#	0.105	−0.058	−0.336#	−0.374#	−0.544#	0.115	−0.257#	0.516#	1.000					
12	HH having BPL Card	0.143*	0.042	0.192#	−0.351#	0.013	−0.398#	−0.191#	0.046	−0.137*	0.071	−0.117*	1.000				
13	SC/ST Population (log)$	−0.025	0.112	0.279#	−0.382#	−0.202#	−0.550#	−0.258#	0.189#	0.006	0.237#	0.142*	0.471#	1.000			
14	HH with Piped Water (log)$	−0.214#	−0.214#	−0.154*	−0.044	−0.320#	−0.395#	−0.530#	0.208#	−0.220#	0.512#	0.691#	−0.109	0.046	1.000		
15	Urban Population	−0.306#	0.058	−0.180#	0.143*	0.038	0.113	−0.317#	0.045	−0.047	0.334#	0.598#	−0.286#	−0.173#	0.574#	1.000	
16	Female literacy	−0.267#	0.007	0.234#	0.107	−0.002	0.164#	−0.394#	0.010	−0.103	0.399#	0.347#	−0.044	−0.128*	0.242#	0.409#	1.000

Note: For full description of variable code, see Table 1.

# Correlation is significant at the 0.01 level (2-tailed).

* Correlation is significant at the 0.05 level (2-tailed).

$ Variables were dropped in the regression model to avoid multicollinearity and for the best fit.

### U5MR and Spatial Clustering

**Figure 1:**
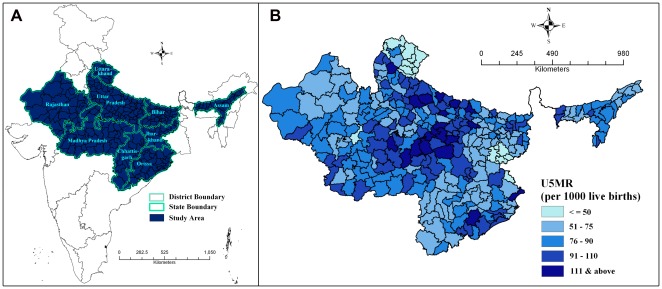
Study area and under-five Mortality. **A.** Location of study area in India **B.** Under-5 Mortality Rate (per 1000 live births) across 284 districts in high focus states of India, 2010–11.

**Figure 1** shows the location of nine high focus states in India (A) and the under-five mortality rate across 284 districts in India's high focus states (B). There were 17 districts in the region, which reported the U5MR below 50 per 1000 live births. However, the number of districts reporting U5MR as 100 and more per 1000 live births was 48, out of which 8 districts namely Kandhmal, Shrawasti, Panna, Satna, Faizabad, Kaushambi, Balrampur, and Chitrakoot (in order of highest to lowest) reported U5MR of 125 and above per 1000 live births.

**Figure 2:**
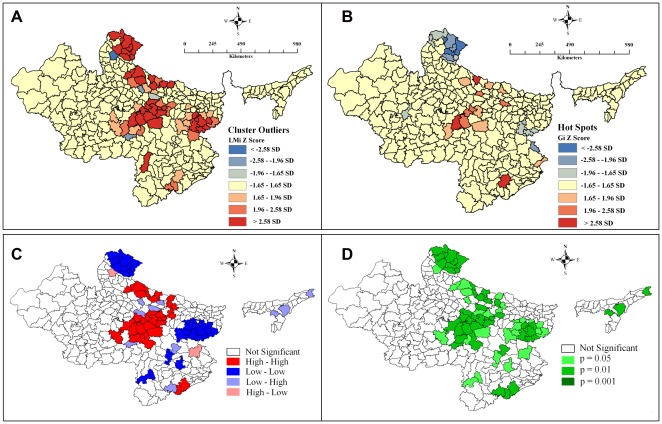
Maps depicting spatial clusters and outliers in under-five mortality rate across 284 districts in high focus states of India, 2010–11. **A.** Cluster and Outlier analysis map (Anseline Local Moran's I = 0.45) using polygon contiguity (first order) weight in ArcGIS. **B.** Hot Spot analysis map (Getis–Ord Gi*) using polygon contiguity (first order) weight in ArcGIS. **C.** Univariate LISA Cluster map (Moran's I = 0.439) using Rook's weight in GeoDa. **D.** Univariate LISA Significance map of Figure C.

**Figure 2** demonstrates the clustering of districts with similar levels of under-five mortality rate using different methods, weights and software. **Figure 2.A** shows the cluster and outlier map using polygon contiguity (first order) weight in ArcGIS. The *Z* score values show statistically significant clusters (red coloured districts) of districts with similar level of U5MR and a few outliers (blue shaded district features). Such clusters are more distinguished in **Figure 2.C**, which demonstrates the univariate LISA cluster map using rook's weight in GeoDa. It distinguishes clearly between a statistically significant (see **Figure 2.D** for significance level) cluster of high values (HH), cluster of low values (LL), outlier in which a high value is surrounded primarily by low values (HL), and outlier in which a low value is surrounded primarily by high values (LH). **Figure 2.B**, on the other hand, demonstrates the Getis–Ord Gi* statistics (*Z* score) using polygon contiguity (first order) weight in ArcGIS. This shows the statistically significant hot spots (clustering of high values) and cold spots (clustering of low values). By assessing selected methods and weights, it is clear that there exist statistically significant clusters in U5MR in the region.

### Ordinary Least Squares (OLS) and Spatial Regression Models

**Figure 3:**
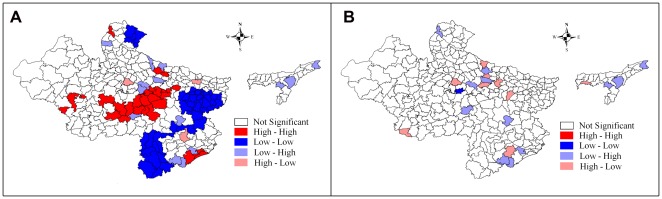
Residual maps of OLS and Spatial Error Model for under-five mortality across 284 districts in high focus states of India, 2010–11. **A.** Univariate LISA Cluster map (Moran's I = 0.416) plotting residuals of OLS regression model. **B.** Univariate LISA Cluster map (Moran's I = 0.200) plotting residuals of Spatial Error regression model.

**Table 4** simulatneously presents the result of an OLS regression model and subsequently employed spatial regression models. Comparing the presented regression diagnostics of OLS and two spatial models, the spatial error (SE) model emerged as the best fit model. However, the Breusch-Pagan test and the Likelihood Ratio test of spatial error dependence are still significant, which indicates that the spatial effects in the data have still not been removed completely. Both spatial models yield improvement to the original OLS model; and the spatial error model appears to be the most improved model. **Figure 3** demonstrates model improvement through residuals maps of OLS and spatial error model for U5MR. The maps show that the problem of spatial autocorrelation amongst the residual terms is largely solved by the spatial error model. The amount of spatial clustering of the residuals is reduced (that is, the residuals appear to be more randomly distributed), and the Moran's *I* of the SE residuals is reduced from 0.416 to 0.200. Imperatively, the highly significant biophysical variables (temperature and rainfall) in the OLS model are no longer significant in the SE model, and female literacy, which was insignificant in the OLS model, is now significant and there is an expected change in sign in the improved model. A detailed explanation on regression diagnostics (including result) of three different multivariate models presented in Table 4 can be accessed in **Appendix S4**.

**Table 4:** Results of OLS model, Spatial Lag model, and Spatial Error model assessing correlates of under-five mortality in high focus states in India, 2010–11.

	Aspatial OLS Model			Spatial Lag Model			Spatial Error Model		
Variable	Coefficient	Std. Error	Probability	Coefficient	Std. Error	Probability	Coefficient	Std. Error	Probability
Urbanization	−0.270	0.089	0.003	−0.292	0.081	0.000	−0.173	0.080	0.030
Population Density (log)	−1.082	1.602	0.500	−2.745	1.463	0.061	0.749	1.919	0.696
Female Literacy	0.006	0.130	0.962	−0.057	0.118	0.629	−0.630	0.158	0.000
HHs having BPL cards	0.191	0.080	0.017	0.110	0.073	0.132	0.296	0.087	0.001
Coverage Gap Index	0.773	0.129	0.000	0.656	0.118	0.000	0.547	0.151	0.000
CHC with LBWM	0.053	0.035	0.137	0.044	0.032	0.169	−0.023	0.026	0.374
Newborn Care provided in PHC (log)	−1.045	0.724	0.150	−1.287	0.656	0.050	−1.370	0.578	0.018
Average Annual Temperature (°C)	2.730	0.498	0.000	1.738	0.463	0.000	−0.563	1.069	0.598
Annual Rainfall (mm)	−0.005	0.002	0.011	−0.003	0.002	0.113	−0.003	0.003	0.209
Constant	−16.610	15.354	0.280	−5.075	13.915	0.715	105.199	29.115	0.000
Number of observations	284			284			284		
Log likelihood	−1191.440			−1168.530			−1114.691		
AIC	2402.870			2359.050			2249.380		
R square	0.368			0.463			0.639		
Lag Coefficient (Rho/Lambda)				0.450	0.050	0.000	1.027	0.001	0.000
Breusch-Pagan test	18.672		0.054	22.796		0.007	21.753		0.010
Likelihood Ratio Test				45.821		0.000	153.489		0.000

In the SE model, the level of urbanization, female literacy, and the number of newborn care provided in Primary Health Centers in the district appeared negatively correlated, and the low economic status (represented by households having BPL cards) as well as the coverge gap in RCH services appeared positively correlated with the incidence of under-five mortality in the region controlling selected biophysical and geographical variables.

### Coverage Gap in RCH Services and U5MR

**Figure 4:**
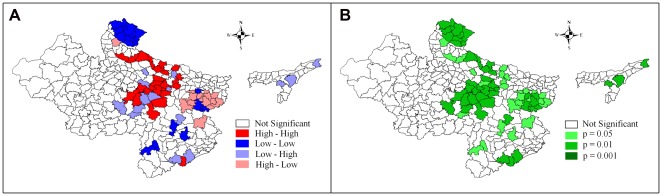
Bivariate LISA (Cluster and Significance) maps depicting spatial clustering and spatial outliers of under-five mortality by coverage gap index across 284 districts in high focus states of India, 2010–11. **A.** Bivariate LISA Cluster map of Under-5 Mortality Rate and Coverage Gap Index. **B.** Bivariate LISA Significance map of Under-5 Mortality Rate and Coverage Gap Index.

Since the coverage gap in a set of RCH services in a district has emerged as a strong and highly significant predictor of under-five mortality, **Figure 4** demonstrates bivariate LISA (cluster and significance) map of CGI and U5MR across 284 districts in high focus states in India. This manifests that the districts with a high coverage gap in RCH services are concurrent with high under-five mortality districts. **Appendix S5** presents the cluster of districts with high U5MR and selected correlates.

### Identifying High Focus District Clusters

**Figure 5:**
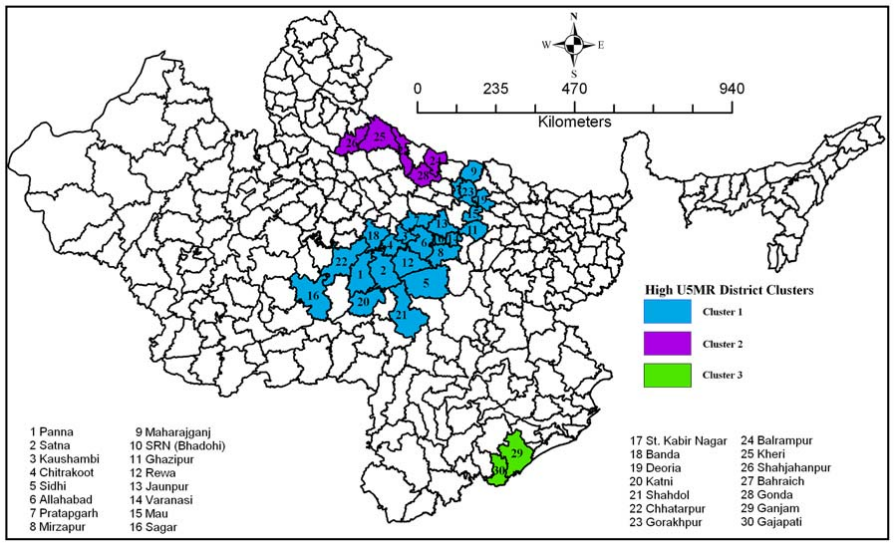
Clusters of districts with high under-five mortality rate.

This study identifies three significant clusters with high U5MR including 30 high focus districts (HFDs) within the high focus states (HFSs) in India (**Figure 5**). Majority of the 30 high focus districts belong to the states of Uttar Pradesh and Madhya Pradesh. However, two districts, namely, Ganjam and Gajapati are located in Orissa. Out of 18 districts, which have under-five mortality above 100 per 1000 live births, 12 are from Uttar Pradesh. Moreover, the cluster of these 12 districts of Uttar Pradesh is located in the eastern part, conventionally believed to be the poor performing regions of the state after Bundelkhand. A similar pattern appears among districts with high under-five mortality in Madhya Pradesh. All high focus group districts in Madhya Pradesh are located in the eastern part and in immediate proximity to the high focus group districts of Uttar Pradesh.

## Discussion

The states in India selected for this study, have been recognized as poor performing states since 1980s, based on their fragile health indicators. However, few studies have covered under-five mortality in this group of states as an area for focused health interventions. This study is the first attempt of its kind to examine factors associated with under-five mortality accounting for biophysical, geographical, health, and socioeconomic variables at the district level. The strength of this study lies in terms of district level coverage, and the spatial analysis method to figure out significant clusters of under-five mortality in selected high focus states in India. Compared to the classical OLS model, the spatial error model (SEM) transpired as an effective and robust model in explaining the predictors of under-five mortality in the selected Indian states, where most of the indicators were spatially auto-correlated. The results of SEM suggest that urbanization, female literacy, households having BPL cards, coverage gap index, and newborn care provided at PHCs were significant correlates of under-five mortality in the study area.

Recent evidences from developing countries have highlighted the importance of potential environmental and geographical correlates in explaining under-five mortality and related health indicators, while adjusting for individual, socioeconomic, household and program level contextual factors [27], [28], [68], [69], [70], [71]. However, this study did not find any direct influence of biophysical and geographical variables in the study area, unlike the results of studies conducted in developing countries like Nepal [26] and Kenya [72]. But the present study supports the view that environmental factors may affect mortality directly; they are more likely to be mediated through individual, socioeconomic and household-level factors such as household income, wealth, education, and other factors [37], [39], [73].

The result suggests that the level of urbanization was negatively associated with under-five mortality in the study area, though a study based on spatio-temporal perspective [29] indicates no significant influence of urbanization on under-five mortality in India. Probably, the effect of urbanization could have been undermined in the study [29], which was based on broader geographical regions rather than administrative divisions like the district. The urban health advantage has been repeatedly emphasized in previous studies irrespective of the world regions [74], [75], [76], [77]. In fact, following maternal education, place of residence (urban/rural contrast) is the most frequent indicator that appears in child survival studies [78], [79] and health research [80], [81]. Urban infrastructure has often been attributed to the improved modern health care system that facilitates public health interventions [82]. Improved electricity, transportation, water and sanitation services are also, on average, more widely available in urban areas than in rural [78]. Infrastructural development including better road and rail links ensures that the urban population receives a fairly regular and abundant supply of services.

One of the most consistent and powerful findings in public health literature is the strong association between mothers' education and child survival [83], [84], [85], [86], [87], [88], [89], [90]. Those studies in developing countries that applied ecological spatial analysis confirm the importance of female literacy as one of the key factors affecting under-five mortality [27]. This finding is particularly imperative for the nine high focus states, as these states have a low level of female literacy [46], [49], [91]. The 2011 census [50] has reported lower literacy among females in six out of the nine high focus states compared to the national average (54%). Moreover, at the national level, the gender gap in education in the 2011 Census was about 22 percentage points, while in Uttar Pradesh and Madhya Pradesh it was 27 and 26 percentage points respectively. In these states, intensified efforts are needed to ensure that young girls are not lagging behind in terms of attaining education. Besides, an effective health-education program is required at the community level to make uneducated women aware of the benefits of timely and appropriate utilization of health services.

The significant effect of households with BPL cards that acts as a surrogate of economic status of population on U5MR is evident in this study. Previous studies in India and elsewhere suggest that poverty or low household income is an important upstream determinant of child survival [92], [93], [94]. It is estimated that more than 200 million children under five years fail to reach their potential in cognitive development because of poverty that has an impact on poor health and nutrition, along with deficient care [95]. Using the third round of NFHS (2005–06) [96], a study reported that household economic status contributed to nearly 46% of under-two mortality at the national level, while it was as high as 76% in Madhya Pradesh [48]. The percentage of population below the poverty line during 2006–07 was higher in four states (Bihar 43%, Orissa 41%, Madhya Pradesh 30%, and Uttar Pradesh 25%) compared to the national average of 19% [97]. The higher mortality among poor households is the outcome of their priority to meet basic daily needs rather than healthcare, whereas wealthier households can spend a higher proportion of their earnings on health care utilization [98], [99]. Moreover, studies have argued that health care programs are more readily accessed by wealthier households [100], [101], while poorer households are excluded because of comparatively higher direct and indirect costs to access health facilities [102].

The study also reveals the significant influence of CGI, which is a summary measure of four health intervention areas on under-five mortality. It is estimated that about half of the child deaths in developing countries can be avoided through adequate coverage of maternal and child health care interventions [103], [104]. There are evidences that show the four health intervention areas, which are included in CGI, have been significantly associated with child survival [11], [46], [105], [106], [107], [108], [109], [110], [111]. The utilization of family planning and maternity services in nine high focus states are below the acceptable level. Estimates show that less than two in every five currently married women were using any contraceptive in Uttar Pradesh, Bihar and Jharkhand [52]. The situation is more bewildering in the case of utilization of full antenatal care (defined as at least three visits for antenatal care check up, at least one Tetanus Toxoid injection received, and consumed 100 iron folic acid tablets/syrup), which ranges from 3%, the lowest in Uttar Pradesh (3%) to the highest in Uttarakhand (16%) [52]. The proportion of women who received full antenatal care was below 5% among 18 out of 30 high focus districts identified in the present study (**Appendix S5**). Additionally, in four out of nine states, there were less than one-fourth of total reproductive age women, who had delivered their last birth in any health facility.

A clear need for effective newborn care to regulate child mortality has emerged in this study which has also been advocated in several studies [109], [112], [113], [114]. Under the NRHM (2005–2012) and the Reproductive and Child Health (RCH) Program Phase-II (2005–10), one of the major components was the establishment of newborn care facilities [115]. Although, about 192 Sick Newborn Care Units (SNCUs), 366 stabilization units, and 1524 newborn care corners have been established, the findings of this study reemphasize the importance of newborn health care facilities in primary healthcare centres (PHCs), especially in the study area. The results of the present study could be considerable for the identified 30 districts with high U5MR in District Health Action Plan (DHAP), recently formulated under the NRHM [116].

### Policy Implications

The results of this study have scope for providing the basis for a few policy implications. Promoting community based education on improved maternal and newborn care, and home-based treatment for newborn infections could enhance child survival in the high priority districts significantly. In addition, effective consultations about specific MCH needs should be provided to women during their first ANC visit in order to ensure the utilization of subsequent maternal and child healthcare services.

In order to cater the nutritional needs of the poor, the Government of India has formulated certain steps by implementing the Mahatma Gandhi National Rural Employment Guarantee Act (MGNREGA) - 2005, National Food Security Bill (NFSB) – 2011, and by strengthening the Public Distribution System (PDS). However, the key question is whether various provisions of the Act are being implemented properly for the desired impact. Some recent studies have shown that the average days of employment provided per beneficiary household under MGNREGA was much lower than the entitled 100 days in most cases, particularly in the districts of the high focus states [117]. Similarly, the poor coverage of PDS in some parts of Bihar, Jharkhand and Uttar Pradesh needs to be addressed effectively for proper distribution of food and non-food items among the poor [118].

The Government of India proposed an increase in public health expenditure up to 3% of GDP and prioritized child health in the Twelfth Five Year Plan 2012–17 [119]. This will involve convergence of health and childcare services. Despite the increase in health budget to around 23% per year in the high focus states during the post-NRHM period, there has to be continuous and high financial support during the Twelfth Five Year Plan. According to the recent Rural Health Statistics 2010 [120], there is a shortage of 19590 Sub-centres; 4252 PHCs and 2115 CHCs in the country. The PHCs in high priority districts need to be upgraded and evaluated periodically, along with quality check. This would require not only infrastructural upgrades but also adequate human resource support and well developed service delivery protocol.

Strengthening and restructuring of the 35-year-old Integrated Child Development Scheme (ICDS), as highlighted in the Twelfth Five Year Plan could play a vital role in ensuring improved child health status in identified clusters of high focus districts. Engaging community participation through *Panchayati Raj* institutions and other stakeholders including representatives from relevant departments like, Women and Child Development, Rural Development, and NGOs would be valuable to ascertain the specific health needs of rural women, their problems in accessing health services, and possible solutions relevant to local districts. In keeping with the initiatives proposed in the NSSK (*Navjaat Shishu Suraksha Karyakram*), this study recognizes the need for strengthening health facilities at the district level to save newborns [115].

In the current circumstances, when several complementary interventions are packaged together and delivered through a range of health-care providers, the main bottlenecks are to ensure improved service delivery including poorly functioning health care system and limited numbers of skilled health-care providers. For this, the present study proposes to develop effective data management as well as a monitoring and surveillance system for assessing key health indicators at the sub-district and block level to identify the hotspots that remain inadequate in health coverage. Additionally, in the last few years, studies have recognized integrating family planning (FP) and maternal and child health (MCH) as a cost-effective way to prevent unintended pregnancies, reduce maternal and child mortality and improve the overall maternal and child health status [121], [122], [123], which could be promoted in high priority districts.

### Limitations of the Study

Although this study examines the effect of biophysical and geographical variables as covariates in the context of under-five mortality analysis in India, we concede a few limitations. First, acknowledging the extent of diversity in the climate, culture, society, economic development, political will, education/awareness and demographic outcomes, the result of this study is limited to the nine high focus states and cannot be generalized in the Indian context entirely. Second, this is a cross-sectional study, which culled data from different sources and the reference period of the indicators ranged from 2007–08 to 2011. Most of the predictor variables included in the analysis represents estimate three or four years prior to the period of the outcome variable, though this can be regarded as one of the strengths of this study. Since the outcome variable itself subsumes a period of more than four years by covering the deaths of children below five years, the prior estimates of predictors would better manifest the lagged effect on the outcome variable; nonetheless, we cannot ignore the possibility of non-congruence among different indicators collected from different sources. Even though all the covariates are district level indicators, the measures, tools of data collection, and coverage of subjects may vary. Third, this study could not use prevalence of undernutrition/malnutrition among children below five years, and causes of death, due to lack of information at the district level. Despite these limitations, the spatial analyses in this study incorporating biophysical and geographical variables exhibit an unexplored dimension in the context of child mortality analysis in India.

## Supporting Information

Appendix S1 **Definition of indicators by intervention area used for the coverage gap index (CGI) at district level.**
(DOC)

Appendix S2 **Discussion on spatial weights and select methods of exploratory spatial data analysis.**
(DOC)

Appendix S3 **Descriptive statistics of variables by 9 high focus states separately.**
(DOC)

Appendix S4 **Detailed explanation on OLS and Spatial Models Diagnostics.**
(DOC)

Appendix S5 **Clusters of districts with a high Under-five Mortality Rate and selected significant indicators.**
(DOC)

## References

[1] United Nations Children's Fund (2010). Levels & Trends in Child Mortality. Estimates Developed by the UN Inter-agency Group for Child Mortality Estimation.

[2] Dyson T, Moore M (1983). On kinship structure, female autonomy, and demographic behavior in India.. Population and Development Review.

[3] Subramanian SV, Nandy S, Irving M, Gordon D, Lambert H (2006). The mortality divide in India: the differential contribution of gender, caste and standard of living across the life course.. American Journal of Public Health.

[4] Arokiasamy P, Pradhan J (2011). Measuring wealth-based health inequality among Indian Children: the importance of equity vs efficiency.. Health Policy and Planning.

[5] Registrar General and Census Commissioner (2012). http://www.censusindia.gov.in/2011-Common/AHSurvey.html.

[6] Lopez A (2000). Reducing child mortality.. Bulletin of the World Health Organization.

[7] Mosley WH, Chen LC (1984). An analytical framework for the study of child survival in developing countries.. Population and Development Review.

[8] Rosenfield A, Min CJ, Freedman LP (2007). Making motherhood safe in developing countries.. New England Journal of Medicine.

[9] Raj A, Saggurti N, Winter M, Labonte A, Decker MR (2010). The effect of maternal child marriage on morbidity and mortality of children under 5 in India: cross sectional study of a nationally representative sample.. British Medical Journal.

[10] Babatunde O (2011). Population and Development.. Science.

[11] Subramanian SV, Ackerson LK, Smith GD, John NA (2009). Association of maternal height with child mortality, anthropometric failure, and anaemia in India.. The Journal of the American Medical Association.

[12] Pathak PK, Singh A (2011). Trends in malnutrition among children in India: growing inequalities across different economic groups.. Social Science and Medicine.

[13] United Nations Children's Fund (2012). http://www.unicef.org/publications/files/Climate_Change_and_Children.pdf.

[14] Paynter S, Ware RS, Weinstein P, Williams G, Sly PD (2010). Childhood Pneumonia: A neglected, climate-sensitive disease?. Lancet.

[15] McMichael AJ, Powels JW, Butler CD, Uauy R (2007). Food, livestock production, energy, climate change, and health.. Lancet.

[16] The World Bank (2008). Environmental health and child survival: epidemiology, economics, experiences.

[17] Martin WJ, Glass RI, Balbus JM, Collins FS (2011). A major environmental cause of death.. Science.

[18] Paul VK, Sachdev HS, Mavalankar D, Ramchandran P, Sankar MJ (2011). Reproductive health, and child health and nutrition in India: meeting the challenge.. Lancet.

[19] Ministry of Health and Family Welfare (2011). Annual Report: 2010–11. Government of India.

[20] Kerber K, de Graft-Johnson JE, Bhutta ZA, Okong P, Starrs A (2007). Continuum of care for maternal, newborn, and child health: from slogan to service delivery.. Lancet.

[21] Shonkoff JP, Richter L, van der Gaag J, Bhutta ZA (2012). An integrated scientific framework for child survival and early childhood development.. Pediatrics.

[22] Kandala NB, Ghilagaber G (2006). A geo-additive Bayesian discrete-time survival model and its application to spatial analysis of childhood mortality in Malawi.. Quality and Quantity.

[23] Goovaerts P, Jacquez GM (2005). Detection of temporal changes in the spatial distribution of cancer rates using LISA statistics and geostatically simulated spatial neutral models.. Journal of Geographical Systems.

[24] Jacquez GM, Greiling DA, Kaufmann A (2005). Design and implementation of space-time information systems.. Journal of Geographical Systems.

[25] Tottrup C, Tersbol BP, Lindeboom W, Meyrowitsch D (2009). Putting child mortality on a map: towards an understanding of inequity in health.. Tropical Medicine and International Health.

[26] Chin B, Montana L, Basgana X (2011). Spatial modeling of geographic inequalities in infant and child mortality across Nepal.. Health and Place.

[27] Sartorious BKD, Sartorious K, Chirwa TF, Fonn S (2011). Infant mortality in South Africa - distribution, associations and policy implications, 2007: an ecological spatial analysis.. International Journal of Health Geographics.

[28] Kazembe LN, Appleton CC, Kleinschmidt I (2007). Spatial analysis of the relationship between early childhood mortality and malaria endemicity in Malawi.. Geospatial Health.

[29] Singh A, Pathak PK, Chauhan RK, Pan W (2011). Infant and child mortality in India in the last two decades: a geospatial analysis.. PLoS ONE.

[30] Demirel R, Erdogan S, Sozen MA (2009). Determination of High Risk Regions of Human Brucellosis in Turkey Using Exploratory Spatial Analysis.. Turkiye Klinikleri Tip Bilimleri Dergisi.

[31] Pouliou T, Elliott SJ (2009). An exploratory spatial analysis of overweight and obesity in Canada.. Preventive Medicine.

[32] Sugumaran R, Larson S, DeGroote J (2009). Spatio-temporal cluster analysis of county-based human West Nile virus incidence in the continental United States.. International Journal of Health Geographics.

[33] Anselin L, Syabri I, Smirnov O, Anselin L, Rey S (2002). Visualizing multivariate spatial correlation with dynamically linked windows..

[34] Waller L, Gotway C (2004). Applied Spatial Statistics for Public Health Data.

[35] Fortin MJ, Dale M (2006). Spatial Analysis: A Guide for Ecologists.

[36] Voss PR, Long DD, Hammer RB, Friedman S (2006). County child poverty rates in the US: A Spatial regression approach.. Population Research and Policy Review.

[37] Balk D, Pullum T, Storeygard A, Greenwell F, Neuman M (2004). A spatial analysis of childhood mortality in West Africa.. Population, Space and Place.

[38] Balk D, Storeygard A, Levy M, Gaskell J, Sharma M (2005). Child hunger in the developing world: An analysis of environmental and social correlates.. Food Policy.

[39] Sherbinin A (2011). The biophysical and geographical correlates of child malnutrition in Africa.. Population, Space and Place.

[40] Webb P (1998). Isolating Hunger: Reaching People in Need Beyond the Mainstream..

[41] Boserup E (1965). The conditions of agricultural growth: The economics of agrarian change under population pressure.

[42] Malthus TR (1798). An essay on the principle of population, as it affects the future improvement in society, with some remarks on the speculations of Mr Godwin, M Condorset, and other writers.

[43] National Research Council (2001). Under the weather: climate, ecosystems, and infectious disease.

[44] Curtis SL, Hossain M (1998). The effect of aridity zone on child nutritional status. West Africa Spatial Analysis Prototype Exploratory Analysis.

[45] Findley S, Balk D, Barlow M, Sogoba N (2002). Putting climate in the service of public health in Mali..

[46] Singh PK, Rai RK, Alagarajan M, Singh L (2012). Determinants of maternity care services utilization among married adolescents in rural India.. PLoS ONE.

[47] Joe W, Mishra US, Navaneetham K (2010). Socio-economic inequalities in child health: Recent evidence from India.. Global Public Health.

[48] Pradhan J, Arokiasamy P (2010). Socio-economic inequalities in child survival in India: a decomposition analysis.. Health Policy.

[49] Arokiasamy P, Gautam A (2008). Neonatal mortality in the empowered action group states of India: trends and determinants.. Journal of Biosocial Science.

[50] Registrar General of India and Census Commissioner (2011). Census of India – 2011: Provisional Population Totals Series 1.

[51] Chandramouli C (2011). Census of India 2011 – a story of innovations..

[52] International Institute for Population Sciences (2010). District Level Household and Facility Survey, 2007–08: India.

[53] Countdown 2008 Equity Analysis Group (2008). Mind the gap: equity and trends in coverage of maternal, newborn, and child health services in 54 Countdown countries.. Lancet.

[54] Warner RM (2008). Applied statistics: from bivariate through multivariate techniques.

[55] Bernard HR (1995). Research methods in anthropology: qualitative and quantitative approaches, 2nd edn.

[56] Planning Commission, Government of India (2012). http://planningcommission.nic.in/news/prmar07.pdf.

[57] MacDougall EB (1992). Exploratory analysis, dynamic statistical visualization, and geographic information systems.. Cartography and Geographic Information Systems.

[58] Moran PAP (1950). Notes on continuous stochastic phenomena.. Biometrika.

[59] Bhattacharjee A, Jensen-Butler C (2005). estimation of spatial weights matrix in a spatial error model, with an application to diffusion in housing demand..

[60] Anselin L, Syabri I, Kho Y (2006). GeoDa: an introduction to spatial data analysis.. Geographical Analysis.

[61] Anselin L (1995). Local indicators of spatial association - LISA.. Geographical Analysis.

[62] Ord JK, Getis A (1995). Local spatial autocorrelation statistics: distributional issues and an application.. Geographical Analysis.

[63] Ord JK, Getis A (2001). Testing for local spatial autocorrelation in the presence of global autocorrelation.. Journal of Regional Science.

[64] Anselin L (1988). Spatial Econometrics: Methods and Models.

[65] Anselin L (1980). Estimation methods for spatial autoregressive structures. Regional Science Dissertation and Monograph Series.

[66] Kelejian HH, Robinson DP, Anselin L, Florax RJ (1995). Spatial correlation: a suggested alternative to the autoregressive model.. New Directions in Spatial Econometrics.

[67] Anselin L, Mills TC, Patterson K (2006). Spatial Econometrics,. Palgrave Handbook of Econometrics: Volume 1, Econometric Theory.

[68] Sartorius BKD, Kahn K, Vounatsou P, Collinson MA, Tollman SM (2010). Young and vulnerable: Spatial-temporal trends and risk factors for infant mortality in rural South Africa (Agincourt), 1992–2007.. BMC Public Health.

[69] Booysen F, Van-Der-Berg S, Burger R, Von Maltitz M, Du Rand G (2008). Using an Asset index to assess trends in poverty in seven sub-Saharan African Countries.. World Development.

[70] Semba RD, Kraemer K, Sun K, de Pee S, Akhter N (2011). Relationship of the presence of a household improved latrine with diarrhoea and under-five child mortality in Indonesia.. American Journal of Tropical Medicine and Hygiene.

[71] Hu Yi, Wang J, Li X, Ren D, Driskell L (2011). Exploring geological and socio-demographic factors associated with under-five mortality in the Wenchuan earthquake using neural network model.. International Journal of Environmental Health Research.

[72] Ombok M, Adazu K, Odhiambo F, Bayoh N, Kiriinya R (2010). Geospatial distribution and determinants of child mortality in rural western Kenya 2002–2005.. Tropical Medicine and International Health.

[73] Lachaud JP (2004). Modeling determinants of child mortality and poverty in the Comoros.. Health and Place.

[74] Fay M, Leipziger D, Wodon Q, Yepes T (2005). Achieving child health related Millenium Development Goals: the role of infrastructure.. World Development.

[75] Firestone R, Punpuing S, Peterson KE, Acevedo-Garcia D, Gortmaker SL (2011). Child overweight and undernutrition in Thailand: is there an urban effect?. Social Science and Medicine.

[76] Fotso JC (2007). Urban–rural differentials in child malnutrition: trends and socioeconomic correlates in sub-Saharan Africa.. Health and Place.

[77] Uzochukwu BSC, Onwujekwe EO, Onoka CA, Ughasoro MD (2008). Rural-urban differences in maternal responses to childhood fever in south east Nigeria.. PLoS ONE.

[78] Sastry N (1997). What explains rural-urban differentials in child mortality in Brazil?. Social Science and Medicine.

[79] Lalou R, Legrand TK (1997). Child mortality in the urban and rural Sahel.. Population.

[80] Harpham T (2009). Urban health in developing countries: what do we know and where do we go?. Health and Place.

[81] Galea S, Vlahov D (2005). Urban health: evidence, challenges and directions.. Annual Review of Public Health.

[82] Fotso JC (2006). Child health inequities in developing countries: difference across urban and rural areas.. International Journal for Equity in Health.

[83] Ozaltin E, Hill K, Subramanian SV (2010). Association of maternal stature with off spring mortality, underweight, and stunting in low- to middle-income countries.. The Journal of the American Medical Association.

[84] Boyle MH, Racine Y, Georqiades K, Snelling D, Hong S (2006). The influence of economic development level, household wealth and maternal education on child health in the developing world.. Social Science and Medicine.

[85] Basu AM, Stephenson R (2005). Low levels of maternal education and the proximate determinants of childhood mortality: a little learning is not a dangerous thing.. Social Science and Medicine.

[86] Kravdal Ø (2004). Child mortality in India: the community-level effect of education.. Population Studies.

[87] Lynch SM (2003). Cohort and life-course patterns in the relationship between education and health: a hierarchical approach.. Demography.

[88] Cleland J (2010). The benefits of educating women.. Lancet.

[89] Cleland JG, Van Ginneken JK (1988). Maternal education and child survival in developing countries: the search for pathways of influence.. Social Science and Medicine.

[90] Gakidou E, Cowling K, Lozano R, Murray CJL (2010). Increased educational attainment and its effect on child mortality in 175 countries between 1970 and 2009: a systematic analysis.. Lancet.

[91] Desai S, Kulkarni V (2008). Changing educational inequalities in India in the context of affirmative action.. Demography.

[92] Claeson M, Bos ER, Mawji T, Pathmanathan I (2000). Reducing child mortality in India in the new millennium.. Bulletin of the World Health Organization.

[93] Sousa A, Hill K, Poz MR (2010). Sub-national assessment of inequality trends in neonatal and child mortality in Brazil.. International Journal for Equity in Health.

[94] Nuwaha F, Babirye J, Oku O, Ayiga N (2011). Understanding socio-economic determinants of childhood mortality: a retrospective analysis in Uganda.. BMC Research Notes.

[95] Grantham-McGreqor S, Cheung YB, Cueto S, Glewwe P, Richter L (2007). Developmental potential in the first 5 years for children in developing countries.. Lancet.

[96] International Institute for Population Sciences & Macro International (2007). National Family Health Survey (NFHS-3), 2005–06: India: Volume I.

[97] Government of India, Indiastat (2012). Incidence of poverty 2006–07.. http://www.indiastat.com/table/economy/8/incidenceofpoverty/221/348040/data.aspx.

[98] Singh L, Rai RK, Singh PK (2012). Assessing the utilization of maternal and child health care among married adolescent women: evidence from India.. Journal of Biosocial Sciences.

[99] Kumar C, Prakash R (2011). Public-private dichotomy in utilization of health care services in India.. Consilience: The Journal of Sustainable Development.

[100] Krishna A (2006). Pathways out of and into poverty in 36 villages of Andhra Pradesh, India.. World Development.

[101] Sen B (2003). Drivers of escape and descent: changing household fortunes in rural Bangladesh.. World Development.

[102] World Health Organisation (2007). Reaching the poor: challenges for child health in the western pacific region. Chapter 7: Barriers to Access to Child Health Care.

[103] Haines A, Sanders D, Lehmann U, Rowe AK, Lawn JE (2007). Achieving child survival goals: potential contribution of community health workers.. Lancet.

[104] Rutherford ME, Dockerty JD, Jasseh M, Howie SRC, Herbison P (2009). Access to health care and mortality of children under 5 years of age in the Gambia: a case–control study.. Bulletin of the World Health Organization.

[105] Boerma JT, Bicego GT (1992). Preceding birth intervals and child survival: searching for pathways of influence.. Studies in Family Planning.

[106] Cleland JG, Sathar ZA (1984). The effect of birth spacing on childhood mortality in Pakistan.. Population Studies.

[107] Dasgupta M (1990). Death clustering, mothers' education and the determinants of child mortality in rural Punjab, India.. Population Studies.

[108] Makepeace G, Pal S (2006). Understanding the Effects of Siblings on Child Mortality: Evidence from India..

[109] Ghosh R (2012). Child mortality in India: a complex situation.. World Journal of Pediatrics.

[110] Ghosh R, Sharma AK (2010). Intra- and inter-household differences in antenatal care, delivery practices and postnatal care between last neonatal deaths and last surviving children in a peri-urban area of India.. Journal of Biosocial Science.

[111] United Nations Children's Fund (2009). The state of Asia-Pacific's children 2008: Child Survival.

[112] Bang AT, Bang RA, Baitule SB, Reddy MH, Deshmukh MD (1999). Effect of home-based neonatal care and management of sepsis on neonatal mortality: field trial in rural India.. Lancet.

[113] Black RE (2009).

[114] Morrison J, Tamang S, Mesko N, Osrin D, Shrestha B (2005). Women's health groups to improve perinatal care in rural Nepal.. BMC Pregnancy Childbirth.

[115] Ministry of Health and Family Welfare (2009). Navjaat Shishu Suraksha Karyakram-2009.

[116] Ministry of Health and Family Welfare (2012). District Health Action Plan Facilitation Guide 2009–10.. http://mohfw.nic.in/NRHM/DHAP.htm.

[117] Haque T (2011). Socio-economic impact of implementation of Mahatma Gandhi National Rural Employment Guarantee Act in India.. Social Change.

[118] Khera R (2011). Revival of the public distribution system: evidence and explanations.. Economic & Political Weekly.

[119] Planning Commission (2012). Faster, Sustainable and More Inclusive Growth: An Approach to the Twelfth Five Year Plan (2012–17).

[120] Ministry of Health and Family Welfare (2012). http://nrhm-mis.nic.in/UI/RHS/RHS%202010/Rural%20Health%20Statistics%202010.htm.

[121] Headey D, Hodge A (2009). The effect of population growth on economic growth: a meta-regression analysis of the macroeconomic literature.. Population and Development Review.

[122] Cecatti JG, Correa-Silva EP, Milanez H, Morais SS, Souza JP (2008). The associations between inter-pregnancy interval and maternal and neonatal outcomes in Brazil.. Maternal and Child Health Journal.

[123] Waddington C, Egger D (2008). Integrated health services—what and why?.

