# Supplementary material for: Under-Five Mortality in High Focus States in India: A District Level Geospatial Analysis
Source: PLoS One. 2012 May 18;7(5):e37515. doi: 10.1371/journal.pone.0037515 (PMC3356406; doi:10.1371/journal.pone.0037515)
Supplement: Appendix S1 — Definition of indicators by intervention area used for the coverage gap index (CGI) at district level. (DOC) [file pone.0037515.s001.doc]

**Appendix S1**

**Table S1.1: Definition of indicators by intervention area used for the coverage gap index (CGI)**

| **Indicators for Coverage Gap Index** | **Definitions** |
| --- | --- |
| ***Indicators for family planning*** |  |
| **Contraceptive prevalence rate** | Percentage of women aged 15–49 years currently married or in union who are using (or whose partner is using) a modern contraceptive method |
| ***Indicators for maternal and newborn care*** |  |
| **Skilled birth attendance** | Percentage of live births in the 3 years before the survey attended by skilled health personnel (doctor, nurse, midwife, or auxiliary midwife) |
| **Antenatal care** | Percentage of women attended at least once during pregnancy by skilled health personnel for reasons related to the pregnancy in the 3 years preceding the survey |
| ***Indicators for immunisation*** |  |
| **Measles vaccination** | Percentage of children aged 12–23 months who are immunised against measles |
| **Diphtheria, pertussis and tetanus vaccination** | Percentage of children aged 12–23 months who received three doses of diphtheria, pertussis, and tetanus vaccine |
| **BCG vaccination** | Percentage of children aged 12–23 months currently vaccinated against BCG |
| ***Indicators for treatment of sick children*** |  |
| **Oral rehydration therapy** | Percentage of children under-5 with diarrhoea in the preceding 2 weeks who received oral rehydration therapy (packets of oral rehydration salts, recommended home solution, or increased fluids) and continued feeding |
| **Treatment of acute respiratory infection** | Percentage of children aged 0–59 months with suspected pneumonia (cough and dyspnoea) who sought care from a health provider |
