# Supplementary material for: Under-Five Mortality in High Focus States in India: A District Level Geospatial Analysis
Source: PLoS One. 2012 May 18;7(5):e37515. doi: 10.1371/journal.pone.0037515 (PMC3356406; doi:10.1371/journal.pone.0037515)
Supplement: Appendix S2 — Discussion on spatial weights and select methods of exploratory spatial data analysis. (DOC) [file pone.0037515.s002.doc]

**Appendix S2**

**Discussion on Spatial Weights and select methods of Exploratory Spatial Data Analysis**

Studies in spatial statistics typically distinguish between two different kinds of spatial effects - spatial interaction (spatial autocorrelation) and spatial structure (spatial heterogeneity). The study of spatial structure is similar to the traditional treatment of coefficient heterogeneity in statistics. Spatial interaction is usually captured by the use of a pre-determined spatial weights matrix. The usual approach to the representation of spatial dependence in cross sectional demographic or other studies is to define a spatial weights matrix, which represents a theoretical and *a priori* understanding of the nature of spatial interdependence between different geographical regions or, more generally, between different demographic/social/economic agents. The spatial weights represent patterns of interaction and diffusion. Thus, the spatial weights provide a meaningful and easily interpretable representation of spatial interaction (spatial autocorrelation) in spatial dependence models [1]. The spatial weights are usually interpreted as functions of relevant measures of demographic/social/economic or geographic distance [2,3]. The distance between two agents reflects their proximity with respect to the unobservable, so that the joint distribution of random variables at a set of points can be represented as a function of the demographic/social/economic distances between them.

The choice of appropriate spatial weights is a central component of spatial models as it imposes *a priori* a structure of spatial dependence, which may or may not correspond to reality. However, the accuracy of these measures profoundly affects the estimation of spatial dependence models [2,4]. The choice typically differs widely across applications, depending not only on specific demographic/social/economic context but also on the availability of data. Spatial contiguity (resting upon implicit assumptions about contagious processes) using a binary representation is a frequent choice. It is often convenient to work with row-standardized spatial weights matrices [5], which are asymmetric by construction. Besides, there are applications where it is reasonable to expect asymmetric strength of diffusion between regions. We opted for contiguity based spatial weights, since our main interest lies in understanding spatial interdependence between the outcome variable and a set of exposure variables in the neighbouring districts. Spatially contiguous weights are generally computed in two ways: (a) rook’s weight (uses common boundaries to define neighbour), and (b) queen’s weight (includes all common points - boundaries and vertices).

ArcGIS calculates polygon contiguity (first order) weight, which is similar to queen’s weight, while GeoDa provides both types of weight. GeoDa is a software tool devised by the Centre for Spatially Integrated Social Sciences (CSISS) to implement various exploratory spatial data analysis including data manipulation, mapping, and spatial regression analysis [6]. We used both the weights to manifest the spatial clustering and outliers in the outcome variable (U5MR) using Anselin Local Moran’s *I* statistics (in ArcGIS) and LISA (Local Indicators of Spatial Autocorrelation, in GeoDa), where ArcGIS used polygon contiguity (first order) or queen’s weight, and rook’s weight was used in GeoDa [7]. Both these local indicators of spatial autocorrelation used in different software are the same, except for their way of presentation and calculation of weights.

**Global Moran’s *I* Index**

Given a set of features and an associated attribute, it evaluates whether the pattern expressed is clustered, dispersed or random. The ArcGIS tool calculates the Moran's *I* Index value [8], and both Z score and p-value evaluating the significance of that index. In general, a Moran's Index value near +1 indicates clustering, while an index value near -1 indicates dispersion. However, without looking at statistical significance, we have no basis to declare if the observed pattern is just one of many possible versions of random.

The Moran’s *I* statistic for spatial autocorrelation is given as:


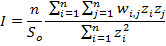


Where z*_i_* is the deviation of an attribute for feature i from its mean
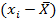
, w*_i,j_* is the spatial weight between feature *i* and *j*, n is equal to the total number of features, and *S_o_* is the aggregate of all the spatial weights:


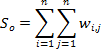


The *z_I_* – score for the statistic is computed as:


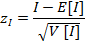


where:


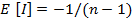


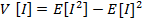


**Local Moran’s *I* statistics (LISA)**

Local spatial autocorrelation statistics provide a measure, for each unit in the region, of the unit’s tendency to have an attribute value that is correlated with values in nearby areas. This computes a measure of spatial association for each individual location.

The Local Moran’s *I* statistic for spatial association is given as:


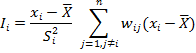


where x*_i_* is an attribute for feature i,
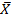
 is the mean of the corresponding attribute, w*_i,j_* is the spatial weight between feature *i* and *j*, and:


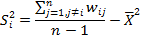


with n equating to the total number of features.

The
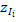
- score for the statistics are computed as:


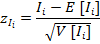


where:


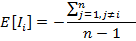


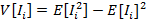


**Bivariate LISA**

Using a similar rationale as in the original development of local indicators of spatial association (LISA) [7], its multivariate generalisation can be defined as follows [9]:


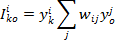


This statistic provides an indication of the degree of linear association (positive or negative) between the values for one variable y_k_ at a given location i, *y^i^_k_* and the average of another variable y_o_ at neighbouring locations j, *y^j^_o_*. A greater than indicated similarity under spatial randomness suggests a spatially similar cluster in the two variables. A dissimilarity greater than spatial randomness would imply a strong, local, negative relationship between the two variables [9]. The significance of the statistic was assessed by means of the permutation approach.

**Getis-Ord Gi* statistics**

The other method to assess spatial clustering in attributes is the hotspot analysis using Getis-Ord Gi* statistic in ArcGIS [10,11]. The resultant *Z* score provides where features with either high or low values cluster spatially. This tool works by looking at each feature within the context of neighbouring features. A feature with a high value is interesting, but may not be a statistically significant hot spot. To be a statistically significant hot spot, a feature will have a high value and that would be surrounded by other features with high values as well. The local sum for a feature and its neighbours is compared proportionally to the sum of all features; when the local sum is much different from the expected local sum, and that difference is too large to be the result of random chance, a statistically significant *Z* score results. For statistically significant positive *Z* scores, the larger the *Z* score is, the more intense the clustering of high values (hot spot). For statistically significant negative *Z* scores, the smaller the *Z* score is, the more intense the clustering of low values (cold spot).

The mathematical formulation of the Getis-Ord Gi* statistic is given as:


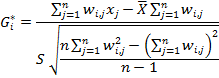


where
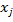
 is the attribute value for feature *j*,
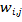
 is the spatial weight between feature *i* and *j*, *n* is equal to the total number of features and:


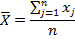


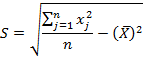


The
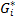
 statistic is a z-score, so no further calculations are required.

**References**

1. Bhattacharjee A, Jensen-Butler C (2005) [estimation of spatial weights matrix in a spatial error model, with an application to diffusion in housing demand](http://ideas.repec.org/p/san/crieff/0519.html). [CRIEFF Discussion Papers](http://ideas.repec.org/s/san/crieff.html) 0519, Centre for Research into Industry, Enterprise, Finance and the Firm.
2. Anselin L (1988) Spatial Econometrics: Methods and Models. Dordrecht: Kluwer Academic Publishers.
3. Anselin L (2002) Under the hood: Issues in the specification and interpretation of spatial regression models. Agricultural Economics 27: 247–67.
4. Fingleton B (2003) Externalities, economic geography and spatial econometrics: conceptual and modeling developments. International Regional Science Review 26: 197–207.
5. Anselin L (1999) Spatial econometrics. In Baltagi BH, ed. A Companion to Theoretical Econometrics. Oxford: Basil Blackwell. pp. 310–330.
6. Anselin L, Syabri I, Kho Y (2006) GeoDa: an introduction to spatial data analysis. Geographical Analysis, 38: 5–22.
7. Anselin L (1995) Local indicators of spatial association - LISA. Geographical Analysis, 27: 93–115.
8. Moran PAP (1950) Notes on continuous stochastic phenomena. Biometrika, 37: 17–23.
9. Anselin L, Syabri I, Smirnov O (2002) Visualizing multivariate spatial correlation with dynamically linked windows. In Anselin L, Rey S, eds. New Tools for Spatial Data Analysis: Proceedings of the Specialist Meeting; Santa Barbara. Center for Spatially Integrated Social Science (CSISS), University of California.
10. Ord JK, Getis A (1995) Local spatial autocorrelation statistics: distributional issues and an application. Geographical Analysis 27: 286–306.
11. Ord JK, Getis A (2001) Testing for local spatial autocorrelation in the presence of global autocorrelation. Journal of Regional Science 41: 411–32.
