# Supplementary material for: Under-Five Mortality in High Focus States in India: A District Level Geospatial Analysis
Source: PLoS One. 2012 May 18;7(5):e37515. doi: 10.1371/journal.pone.0037515 (PMC3356406; doi:10.1371/journal.pone.0037515)
Supplement: Appendix S3 — Descriptive statistics of variables by 9 high focus states separately. (DOC) [file pone.0037515.s003.doc]

**Appendix S3**

**Descriptive Statistics of Variables by High Focus States**

**Table S3.1.** Descriptive Statistics of Variables, Assam

| **Variables** | **Minimum** | **Maximum** | **Mean** | **Std. Deviation** |
| --- | --- | --- | --- | --- |
| U5MR (per 1000 live births) | 52 | 103 | 78 | 12 |
| Average Annual Temperature (°C) | 21.7 | 24.9 | 24.0 | 0.8 |
| Annual Rainfall (mm) | 1053 | 3285 | 2107.6 | 639.9 |
| Foodgrain Yield (quintal/hectare) | 8.1 | 18.7 | 14.0 | 2.5 |
| % Area of District within 2 km from Road | 17.8 | 38.7 | 26.4 | 6.0 |
| Population Density (person/sq. km) | 44 | 1171 | 467 | 237 |
| Coverage Gap Index [CGI] | 39.5 | 67.9 | 56.1 | 6.9 |
| % CHC with Low Birth Weight Management | 0.0 | 100.0 | 46.1 | 31.4 |
| Number of newborn care provided in PHC | 69 | 675 | 236 | 148 |
| Knowledge about Malaria Prevention | 76.5 | 97.3 | 91.2 | 5.3 |
| Per Capita GDDP (in INR) | 8892 | 25215 | 16582 | 4946 |
| % HH having BPL Card | 8.9 | 31.6 | 21.2 | 6.5 |
| % SC/ST Population | 10.8 | 71.4 | 38.2 | 17.1 |
| % HH with Piped Water | 0.6 | 33.0 | 5.4 | 8.0 |
| Female literacy (%) | 54.3 | 78.2 | 67.7 | 6.5 |
| % Urban Population | 6.1 | 28.7 | 12.0 | 5.6 |
| N = 23 (Districts) |  |  |  |  |

**Table S3.2.** Descriptive Statistics of Variables, Bihar

| **Variables** | **Minimum** | **Maximum** | **Mean** | **Std. Deviation** |
| --- | --- | --- | --- | --- |
| U5MR (per 1000 live births) | 53 | 106 | 77 | 13 |
| Average Annual Temperature (°C) | 24.6 | 26.6 | 25.6 | 0.7 |
| Annual Rainfall (mm) | 402.6 | 1850.3 | 1009.3 | 309.8 |
| Foodgrain Yield (quintal/hectare) | 9.0 | 25.9 | 17.3 | 4.5 |
| % Area of District within 2 km from Road | 31.9 | 51.6 | 40.3 | 5.1 |
| Population Density (person/sq. km) | 484 | 1803 | 1124 | 339 |
| Coverage Gap Index [CGI] | 49.5 | 75.7 | 64.2 | 6.2 |
| % CHC with Low Birth Weight Management | 0.0 | 100.0 | 24.8 | 36.4 |
| Number of newborn care provided in PHC | 35 | 3927 | 1246 | 827 |
| Knowledge about Malaria Prevention | 8.8 | 97.0 | 59.5 | 28.9 |
| Per Capita GDDP (in INR) | 3636 | 31441 | 6584 | 4392 |
| % HH having BPL Card | 12.8 | 44.7 | 26.4 | 6.1 |
| % SC/ST Population | 11.2 | 35.3 | 22.8 | 5.1 |
| % HH with Piped Water | 0.0 | 18.5 | 1.2 | 3.1 |
| Female literacy (%) | 42.7 | 65.5 | 53.1 | 6.7 |
| % Urban Population | 3.5 | 43.5 | 10.7 | 7.7 |
| N = 23 (Districts) |  |  |  |  |

**Table S3.3.** Descriptive Statistics of Variables, Chhattisgarh

| **Variables** | **Minimum** | **Maximum** | **Mean** | **Std. Deviation** |
| --- | --- | --- | --- | --- |
| U5MR (per 1000 live births) | 52 | 103 | 72 | 14 |
| Average Annual Temperature (°C) | 23.5 | 27.3 | 25.8 | 1.1 |
| Annual Rainfall (mm) | 637.3 | 1405.8 | 1077.8 | 178.7 |
| Foodgrain Yield (quintal/hectare) | 11.3 | 17.9 | 14.9 | 2.0 |
| % Area of District within 2 km from Road | 33.7 | 43.9 | 37.7 | 3.1 |
| Population Density (person/sq. km) | 45 | 421 | 212 | 117 |
| Coverage Gap Index [CGI] | 39.7 | 59.6 | 50.1 | 7.1 |
| % CHC with Low Birth Weight Management | 20.0 | 100.0 | 49.5 | 24.6 |
| Number of newborn care provided in PHC | 44 | 224 | 141 | 59 |
| Knowledge about Malaria Prevention | 76.0 | 95.1 | 89.7 | 5.4 |
| Per Capita GDDP (in INR) | 8039 | 56029 | 16094 | 11159 |
| % HH having BPL Card | 40.9 | 69.6 | 56.8 | 8.2 |
| % SC/ST Population | 28.6 | 79.2 | 51.3 | 15.5 |
| % HH with Piped Water | 1.4 | 14.2 | 7.0 | 4.2 |
| Female literacy (%) | 32.2 | 70.5 | 58.5 | 10.2 |
| % Urban Population | 8.9 | 38.4 | 19.8 | 10.4 |
| N = 16 (Districts) |  |  |  |  |

**Table S3.4.** Descriptive Statistics of Variables, Jharkhand

| **Variables** | **Minimum** | **Maximum** | **Mean** | **Std. Deviation** |
| --- | --- | --- | --- | --- |
| U5MR (per 1000 live births) | 36 | 93 | 62 | 18 |
| Average Annual Temperature (°C) | 23.4 | 26.6 | 25.2 | 0.8 |
| Annual Rainfall (mm) | 333.6 | 1484.9 | 963.5 | 274.1 |
| Foodgrain Yield (quintal/hectare) | 7.6 | 20.3 | 11.0 | 3.2 |
| % Area of District within 2 km from Road | 22.3 | 52.7 | 33.0 | 7.2 |
| Population Density (person/sq. km) | 179 | 1307 | 502 | 259 |
| Coverage Gap Index [CGI] | 42.8 | 71.4 | 59.6 | 7.8 |
| % CHC with Low Birth Weight Management | 0.0 | 50.0 | 2.8 | 11.8 |
| Number of newborn care provided in PHC | 22 | 598 | 192 | 187 |
| Knowledge about Malaria Prevention | 57.5 | 94.9 | 82.5 | 9.2 |
| Per Capita GDDP (in INR) | 8054 | 22780 | 14112 | 4219 |
| % HH having BPL Card | 17.1 | 50.2 | 31.3 | 8.9 |
| % SC/ST Population | 20.5 | 76.7 | 45.6 | 16.7 |
| % HH with Piped Water | 0.2 | 34.3 | 6.7 | 9.8 |
| Female literacy (%) | 41.2 | 67.3 | 54.1 | 7.2 |
| % Urban Population | 4.9 | 58.1 | 20.2 | 17.6 |
| N = 18 (Districts) |  |  |  |  |

**Table S3.5.** Descriptive Statistics of Variables, Madhya Pradesh

| **Variables** | **Minimum** | **Maximum** | **Mean** | **Std. Deviation** |
| --- | --- | --- | --- | --- |
| U5MR (per 1000 live births) | 51 | 140 | 90 | 17 |
| Average Annual Temperature (°C) | 23.4 | 26.6 | 25.4 | 0.7 |
| Annual Rainfall (mm) | 540.8 | 1314.5 | 889.2 | 167.1 |
| Foodgrain Yield (quintal/hectare) | 4.6 | 29.7 | 11.7 | 5.6 |
| % Area of District within 2 km from Road | 18.3 | 46.6 | 32.4 | 7.1 |
| Population Density (person/sq. km) | 94 | 854 | 261 | 152 |
| Coverage Gap Index [CGI] | 32.5 | 67.8 | 51.4 | 8.2 |
| % CHC with Low Birth Weight Management | 0.0 | 100.0 | 51.2 | 28.8 |
| Number of newborn care provided in PHC | 25 | 4437 | 571 | 888 |
| Knowledge about Malaria Prevention | 58.6 | 98.0 | 90.1 | 7.7 |
| Per Capita GDDP (in INR) | 8370 | 31677 | 13464 | 4679 |
| % HH having BPL Card | 19.6 | 62.9 | 42.0 | 10.2 |
| % SC/ST Population | 17.0 | 83.0 | 38.5 | 15.8 |
| % HH with Piped Water | 0.5 | 34.2 | 10.1 | 7.2 |
| Female literacy (%) | 32.7 | 76.6 | 59.0 | 8.4 |
| % Urban Population | 4.6 | 80.8 | 24.7 | 15.6 |
| N = 45 (Districts) |  |  |  |  |

**Table S3.6.** Descriptive Statistics of Variables, Orissa

| **Variables** | **Minimum** | **Maximum** | **Mean** | **Std. Deviation** |
| --- | --- | --- | --- | --- |
| U5MR (per 1000 live births) | 58 | 145 | 82 | 19 |
| Average Annual Temperature (°C) | 24.0 | 27.3 | 26.3 | 0.9 |
| Annual Rainfall (mm) | 1276.2 | 1667.6 | 1451.4 | 110.8 |
| Foodgrain Yield (quintal/hectare) | 8.0 | 17.7 | 12.1 | 2.5 |
| % Area of District within 2 km from Road | 24.1 | 41.2 | 34.2 | 4.3 |
| Population Density (person/sq. km) | 91 | 799 | 317 | 207 |
| Coverage Gap Index [CGI] | 33.9 | 66.3 | 47.2 | 8.6 |
| % CHC with Low Birth Weight Management | 0.0 | 85.7 | 36.4 | 27.3 |
| Number of newborn care provided in PHC | 0 | 508 | 155 | 152 |
| Knowledge about Malaria Prevention | 33.4 | 98.2 | 84.7 | 13.9 |
| Per Capita GDDP (in INR) | 9472 | 43130 | 15218 | 7008 |
| % HH having BPL Card | 32.7 | 67.9 | 51.9 | 9.3 |
| % SC/ST Population | 17.2 | 86.5 | 47.6 | 19.7 |
| % HH with Piped Water | 0.7 | 16.9 | 4.8 | 4.5 |
| Female literacy (%) | 37.2 | 82.1 | 62.1 | 14.0 |
| % Urban Population | 4.6 | 48.1 | 14.8 | 10.9 |
| N = 30 (Districts) |  |  |  |  |

**Table S3.7.** Descriptive Statistics of Variables, Rajasthan

| **Variables** | **Minimum** | **Maximum** | **Mean** | **Std. Deviation** |
| --- | --- | --- | --- | --- |
| U5MR (per 1000 live births) | 45 | 99 | 79 | 10 |
| Average Annual Temperature (°C) | 24.2 | 27.1 | 25.7 | 0.6 |
| Annual Rainfall (mm) | 246.4 | 972.5 | 531.6 | 190.6 |
| Foodgrain Yield (quintal/hectare) | 1.7 | 29.9 | 15.5 | 7.8 |
| % Area of District within 2 km from Road | 20.0 | 41.0 | 29.6 | 4.4 |
| Population Density (person/sq. km) | 17 | 598 | 265 | 132 |
| Coverage Gap Index [CGI] | 35.3 | 62.5 | 48.2 | 7.2 |
| % CHC with Low Birth Weight Management | 0.0 | 71.4 | 35.5 | 18.2 |
| Number of newborn care provided in PHC | 134 | 1538 | 329 | 253 |
| Knowledge about Malaria Prevention | 72.2 | 99.6 | 91.4 | 7.3 |
| Per Capita GDDP (in INR) | 11048 | 24537 | 17266 | 3933 |
| % HH having BPL Card | 8.8 | 41.1 | 19.3 | 7.7 |
| % SC/ST Population | 17.2 | 79.3 | 32.3 | 14.2 |
| % HH with Piped Water | 7.1 | 58.6 | 21.0 | 12.4 |
| Female literacy (%) | 38.7 | 66.3 | 50.9 | 7.0 |
| % Urban Population | 6.4 | 60.3 | 22.1 | 11.8 |
| N = 32 (Districts) |  |  |  |  |

**Table S3.8.** Descriptive Statistics of Variables, Uttar Pradesh

| **Variables** | **Minimum** | **Maximum** | **Mean** | **Std. Deviation** |
| --- | --- | --- | --- | --- |
| U5MR (per 1000 live births) | 52 | 142 | 94 | 19 |
| Average Annual Temperature (°C) | 24.0 | 26.2 | 25.4 | 0.5 |
| Annual Rainfall (mm) | 209.0 | 1338.2 | 597.0 | 213.4 |
| Foodgrain Yield (quintal/hectare) | 3.8 | 34.5 | 22.1 | 6.3 |
| % Area of District within 2 km from Road | 26.6 | 51.3 | 38.4 | 5.9 |
| Population Density (person/sq. km) | 242 | 2383 | 909 | 406 |
| Coverage Gap Index [CGI] | 51.0 | 79.2 | 65.3 | 6.3 |
| % CHC with Low Birth Weight Management | 0.0 | 100.0 | 25.5 | 25.6 |
| Number of newborn care provided in PHC | 11 | 1305 | 148 | 183 |
| Knowledge about Malaria Prevention | 46.9 | 94.7 | 73.0 | 12.6 |
| Per Capita GDDP (in INR) | 5215 | 47973 | 12131 | 6163 |
| % HH having BPL Card | 4.3 | 49.3 | 27.3 | 12.2 |
| % SC/ST Population | 9.6 | 38.8 | 21.1 | 7.1 |
| % HH with Piped Water | 0.2 | 32.7 | 6.3 | 6.5 |
| Female literacy (%) | 37.1 | 81.4 | 58.7 | 8.2 |
| % Urban Population | 3.4 | 67.5 | 20.6 | 15.2 |
| N = 70 (Districts) |  |  |  |  |

**Table S3.9.** Descriptive Statistics of Variables, Uttarakhand

| **Variables** | **Minimum** | **Maximum** | **Mean** | **Std. Deviation** |
| --- | --- | --- | --- | --- |
| U5MR (per 1000 live births) | 24 | 94 | 45 | 20 |
| Average Annual Temperature (°C) | 9.6 | 24.3 | 16.6 | 4.3 |
| Annual Rainfall (mm) | 943.0 | 2271.8 | 1417.3 | 395.1 |
| Foodgrain Yield (quintal/hectare) | 7.7 | 31.0 | 14.8 | 6.9 |
| % Area of District within 2 km from Road | 0.2 | 45.7 | 25.4 | 12.2 |
| Population Density (person/sq. km) | 41 | 817 | 245 | 254 |
| Coverage Gap Index [CGI] | 38.5 | 55.0 | 44.1 | 5.1 |
| % CHC with Low Birth Weight Management | 0.0 | 66.7 | 36.6 | 22.2 |
| Number of newborn care provided in PHC | 0 | 163 | 47 | 64 |
| Knowledge about Malaria Prevention | 66.1 | 88.4 | 77.4 | 5.9 |
| Per Capita GDDP (in INR) | 13532 | 31245 | 20576 | 4945 |
| % HH having BPL Card | 20.1 | 47.9 | 33.7 | 10.2 |
| % SC/ST Population | 13.4 | 34.0 | 21.8 | 5.7 |
| % HH with Piped Water | 19.4 | 73.0 | 32.8 | 14.0 |
| Female literacy (%) | 46.7 | 71.2 | 58.7 | 7.5 |
| % Urban Population | 3.5 | 55.9 | 20.4 | 16.2 |
| N = 13 (Districts) |  |  |  |  |
