# Supplementary material for: Under-Five Mortality in High Focus States in India: A District Level Geospatial Analysis
Source: PLoS One. 2012 May 18;7(5):e37515. doi: 10.1371/journal.pone.0037515 (PMC3356406; doi:10.1371/journal.pone.0037515)
Supplement: Appendix S4 — Detailed explanation on OLS and Spatial Models Diagnostics. (DOC) [file pone.0037515.s004.doc]

**Appendix S4**

**Detailed Explanation on OLS and Spatial Models Diagnostics**

First, we concetrate on the OLS model and its fit under classical BLUE (Best Linear Unbiased Estimator) assumptions. In the OLS model, the level of urbanization, households having BPL cards, coverage gap index, and both the biophysical variables (temperature and rainfall) appeared as significant correlates of the under-five mortality rate in the selected region. However, it would be early to conclude the result here before examining the other diagnostics of the model. Given the Jarque-Bera test score (2.763; *p*=0.251), which indicates the normal distribution of error term, the low probability of Breusch-Pagan test points to the existence of heteroskedasticity. This is not necessarily a surprise because the error variance could well be affected by the spatial dependence in the data. Moran’s *I* score of 0.416 (*p*<0.001) is highly significant, indicating a strong spatial autocorrelation of the residuals. In addition, both Lagrange Multiplier (lag) and Lagrange Multiplier (error) are significant, indicating the presence of spatial dependence. The robust measure for error (74.709; *p*<0.001) is still significant, but the robust lag test (0.682; *p*=0.409) becomes insignificant, which means that when a lagged dependent variable is present, the error dependence disappears. The AIC (Akaike Information Criterion) is a measure of the relative [goodness of fit](http://en.wikipedia.org/wiki/Goodness_of_fit) of a [statistical model](http://en.wikipedia.org/wiki/Statistical_model) [1]. In the general case, AIC = 2*k* – 2ln(*L*); where *k* is the number of [parameters](http://en.wikipedia.org/wiki/Parameter) in the [statistical model](http://en.wikipedia.org/wiki/Statistical_model), and *L* is the maximized value of the [likelihood function](http://en.wikipedia.org/wiki/Likelihood_function) for the estimated model. Given a set of models for the data, the preferred model is the one with the minimum AIC value. Moreover, the model coefficient of determination (R-squared = 0.368) also indicates that the OLS model is not a best fit for this group of data.

After identifying the presence of spatial dependence, the spatial lag model was applied with maximum likelihood approach [2], the result of which is presented in Table 4. The spatial lag coefficient (Rho‘*ρ*’) of U5MR appeared as an additional indicator in the model. The coefficient parameter (*ρ*) reflects the spatial dependence inherent in the sample data, measuring the average influence on observations by their neighbouring observations. It has a positive effect and is highly significant. As a result, the general model fit improved, as indicated in the higher values of R-squared and Log likelihood. The effects of other independent variables remain virtually the same. However, the significance of Breusch-Pagan test and the Likelihood Ratio test of spatial lag dependence suggests that although the introduction of spatial lag term improved the model fit, it did not make the spatial effects go away.

We, then, employed the spatial error model. Compared to spatial lag model’s Rho (*ρ*), we have now a coefficient on the spatially correlated errors (Lambda ‘*λ*’) as an additional indicator. It has a positive effect and is highly significant too. As a result, the general model fit improved, as indicated in higher values of R-squared and Log Likelihood. The Breusch-Pagan test and the Likelihood Ratio test of spatial error dependence are still significant, which indicates that the spatial effects in the data have still not been removed completely. However, both spatial models yield improvement to the original OLS model; the spatial error model appears to be the most improved model among all. Figure 3 demonstrates model improvement through residuals maps of OLS and spatial error model for U5MR. The maps show that the problem of spatial autocorrelation amongst the residual terms is largely solved by the spatial error model. The amount of spatial clustering of the residuals is reduced (that is, the residuals appear to be more randomly distributed), and the Moran’s *I* of the SE residuals is reduced from 0.416 to 0.200.


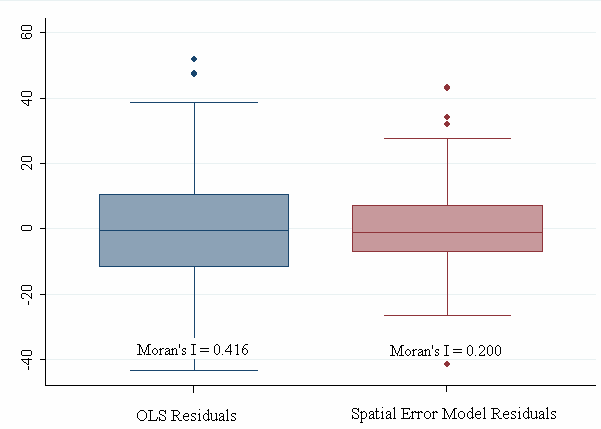


**Figure S3.1.** Boxplot and Moran’s *I* of OLS residuals and Spatial Error Model residuals for under-five mortality across 284 districts in high focus states of India, 2010-11.

**Figure S3.1** shows that the spread of the residuals is substantially reduced in the SE model when compared to the OLS model, which is another sign that the SE model has succeeded in correcting for spatial autocorrelation amongst the variables. Importantly, the highly significant biophysical variables (temperature and rainfall) in the OLS model are no longer significant in the SE model, and female literacy, which was insignificant in the OLS model, is now significant and there is an expected change in sign in the improved model.

In the SE model, the level of urbanization, female literacy, and the number of newborn care provided in Primary Health Centers in the district appeared as negatively correlated, and the low economic status (represented by households having BPL cards) as well as the coverge gap in RCH services appeared as positively correlated with the incidence of under-five mortality in the region controlling selected biophysical and geographical variables.

**References**

1. [Akaike H](http://en.wikipedia.org/wiki/Hirotsugu_Akaike) (1974) A new look at the statistical model identification. IEEE Transactions on Automatic Control 19: 716–23.
2. Anselin L (2005) Exploring spatial data with GeoDa: A workbook. Center for Spatially Integrated Social Science. Available: [http://www.csiss.org](http://www.csiss.org/). Accessed 20 December 2011.
