# Supplementary material for: Under-Five Mortality in High Focus States in India: A District Level Geospatial Analysis
Source: PLoS One. 2012 May 18;7(5):e37515. doi: 10.1371/journal.pone.0037515 (PMC3356406; doi:10.1371/journal.pone.0037515)
Supplement: Appendix S5 — Clusters of districts with a high Under-five Mortality Rate and selected significant indicators. (DOC) [file pone.0037515.s005.doc]

**Appendix S5**

**Table S5.1: Clusters of districts with a high under-five mortality rate and selected significant indicators.**

| Districts | U5MR | IMR | CGI | Female Literacy | % Urban | % HH having BPL Card | Per Capita GDP | ORT | ARI | CPR | SBA | Full ANC | MSL | DPT3 | BCG | NBC PHC |
| --- | --- | --- | --- | --- | --- | --- | --- | --- | --- | --- | --- | --- | --- | --- | --- | --- |
| **Cluster 1** |  |  |  |  |  |  |  |  |  |  |  |  |  |  |  |  |
| Panna | 140 | 93 | 58.9 | 55.6 | 12.3 | 55.6 | 10604 | 23.8 | 47.8 | 51.5 | 42.7 | 6.9 | 55.1 | 37.0 | 80.3 | 1439 |
| Satna | 130 | 90 | 54.5 | 63.4 | 21.3 | 54.6 | 12403 | 46.9 | 55.8 | 49.1 | 46.5 | 4.8 | 54.7 | 42.3 | 84.7 | 295 |
| Kaushambi | 128 | 83 | 74.1 | 50.8 | 7.8 | 24.0 | 10467 | 17.9 | 45.0 | 22.7 | 20.9 | 3.5 | 35.6 | 24.6 | 63.6 | 60 |
| Chitrakoot | 125 | 69 | 67.0 | 54.0 | 9.7 | 33.8 | 7260 | 32.4 | 49.7 | 33.8 | 24.7 | 1.8 | 44.1 | 29.9 | 71.9 | 16 |
| Sidhi | 118 | 72 | 67.8 | 52.6 | 13.9 | 48.9 | 19509 | 5.0 | 54.3 | 38.9 | 27.2 | 4.6 | 47.1 | 29.8 | 70.2 | 286 |
| Allahabad | 115 | 88 | 65.6 | 62.7 | 24.8 | 15.8 | 12034 | 32.3 | 50.9 | 35.7 | 34.2 | 5.6 | 40.3 | 32.3 | 56.7 | 120 |
| Pratapgarh | 113 | 88 | 58.2 | 61.0 | 5.5 | 32.3 | 6452 | 28.0 | 67.2 | 24.2 | 44.4 | 7.6 | 63.9 | 63.9 | 86.4 | 83 |
| Mirzapur | 111 | 83 | 65.5 | 58.8 | 13.9 | 34.2 | 8749 | 21.1 | 55.1 | 40.1 | 39.2 | 4.3 | 33.3 | 31.9 | 54.2 | 42 |
| Maharajganj | 110 | 87 | 64.6 | 50.1 | 5.1 | 40.7 | 6960 | 16.1 | 69.4 | 31.1 | 19.5 | 3.5 | 49.4 | 45.2 | 85.7 | 66 |
| SRN (Bhadohi) | 109 | 80 | 67.3 | 57.8 | 14.7 | 31.7 | 10585 | 19.6 | 63.7 | 29.3 | 34.6 | 2.6 | 36.2 | 32.4 | 64.6 | 192 |
| Ghazipur | 102 | 82 | 64.9 | 62.3 | 7.6 | 33.3 | 7557 | 13.6 | 66.3 | 25.3 | 37.0 | 3.7 | 57.9 | 43.5 | 74.3 | 101 |
| Rewa | 102 | 73 | 55.0 | 62.5 | 16.7 | 42.0 | 10100 | 36.8 | 53.1 | 47.1 | 48.1 | 5.2 | 64.4 | 47.3 | 87.0 | 611 |
| Jaunpur | 101 | 78 | 63.2 | 61.7 | 7.5 | 33.3 | 6829 | 14.3 | 54.9 | 31.1 | 44.5 | 4.9 | 47.5 | 52.5 | 73.9 | 62 |
| Varanasi | 101 | 78 | 51.0 | 68.2 | 43.4 | 19.8 | 11377 | 36.8 | 69.4 | 43.6 | 54.6 | 3.5 | 64.5 | 64.5 | 87.0 | 110 |
| Mau | 97 | 76 | 61.9 | 65.6 | 22.7 | 38.0 | 9647 | 20.0 | 61.6 | 22.5 | 48.1 | 6.4 | 64.5 | 49.4 | 83.9 | 76 |
| Sagar | 97 | 70 | 53.2 | 67.7 | 29.8 | 52.8 | 11732 | 36.8 | 54.8 | 52.7 | 49.8 | 13.4 | 68.0 | 36.5 | 87.3 | 467 |
| Sant Kabir Nagar | 97 | 65 | 65.5 | 57.0 | 7.5 | 48.5 | 6971 | 17.6 | 68.9 | 17.4 | 28.4 | 3.9 | 58.4 | 53.8 | 78.3 | 119 |
| Banda | 95 | 60 | 69.2 | 55.0 | 15.3 | 31.2 | 9728 | 24.4 | 65.6 | 28.9 | 24.0 | 3.4 | 35.0 | 24.5 | 58.7 | 64 |
| Deoria | 93 | 75 | 56.2 | 61.3 | 10.2 | 41.2 | 6424 | 18.0 | 74.3 | 26.6 | 46.6 | 6.0 | 63.9 | 73.0 | 95.1 | 272 |
| Katni | 93 | 70 | 52.5 | 62.5 | 20.4 | 42.2 | 15155 | 40.0 | 41.7 | 50.1 | 51.3 | 6.2 | 74.0 | 58.9 | 88.5 | 646 |
| Shahdol | 92 | 77 | 59.1 | 58.1 | 23.4 | 53.8 | 15188 | 15.4 | 35.0 | 49.0 | 45.2 | 12.7 | 64.3 | 44.7 | 88.2 | 4437 |
| Chhatarpur | 89 | 72 | 60.5 | 54.3 | 22.6 | 33.2 | 10881 | 16.7 | 54.1 | 45.3 | 54.1 | 4.2 | 51.3 | 29.6 | 82.6 | 381 |
| Gorakhpur | 82 | 61 | 56.1 | 61.5 | 18.8 | 34.6 | 9715 | 28.6 | 75.3 | 32.1 | 36.5 | 9.1 | 60.3 | 62.1 | 90.2 | 60 |
| **Cluster 2** |  |  |  |  |  |  |  |  |  |  |  |  |  |  |  |  |
| Balrampur | 128 | 93 | 78.1 | 40.9 | 7.7 | 29.0 | 8162 | 15.0 | 68.6 | 7.0 | 10.7 | 2.0 | 24.4 | 25.3 | 54.6 | 11 |
| Kheri | 117 | 79 | 71.2 | 52.6 | 11.5 | 38.4 | 11033 | 26.1 | 71.6 | 23.4 | 17.8 | 1.6 | 30.9 | 22.2 | 57.0 | 312 |
| Shahjahanpur | 106 | 87 | 73.0 | 51.7 | 19.7 | 16.2 | 10992 | 16.7 | 68.5 | 20.2 | 11.9 | 1.2 | 39.5 | 25.8 | 62.9 | 246 |
| Bahraich | 101 | 66 | 79.2 | 40.8 | 8.3 | 43.8 | 7004 | 7.2 | 55.1 | 8.8 | 11.3 | 2.6 | 30.3 | 23.8 | 67.3 | 42 |
| Gonda | 97 | 72 | 71.5 | 49.1 | 6.6 | 25.7 | 7481 | 28.1 | 69.6 | 12.5 | 24.7 | 1.3 | 32.0 | 30.6 | 64.6 | 26 |
| **Cluster 3** |  |  |  |  |  |  |  |  |  |  |  |  |  |  |  |  |
| Ganjam | 93 | 61 | 50.6 | 61.8 | 21.8 | 45.6 | 12216 | 43.2 | 65.2 | 38.3 | 58.0 | 19.3 | 64.2 | 59.3 | 83.5 | 508 |
| Gajapati | 85 | 65 | 66.3 | 43.6 | 12.2 | 44.0 | 12604 | 0.0 | 25.0 | 33.8 | 25.3 | 23.7 | 62.5 | 58.3 | 77.1 | 264 |
